# Targeting childhood loneliness in china: *in silico* interventions and moderated network analysis

**DOI:** 10.1186/s13034-025-00947-9

**Published:** 2025-07-26

**Authors:** Xinle Yu, Xuanzhi Zhang, Kusheng Wu, Zhenqiang Xu, Zhiya Liang, Wanyi Wen, Dinghui Wang, Yanhong Huang

**Affiliations:** 1 Mental Health Center of Shantou University, Shantou, Guangdong China; 2 Shantou University Medical College—Faculty of Medicine of University of Manitoba Joint Laboratory of Biological Psychiatry, Shantou, Guangdong China; 3 Department of Preventive Medicine, Shantou University Medical College, Shantou, Guangdong China; 4 School of Public Health, Zhaoqing Medical College, Zhaoqing, Guangdong China; 5 The People’s Hospital of Gaoming District of Foshan City, Foshan, Guangdong China

**Keywords:** Childhood loneliness, *In silico* intervention, Moderated network analysis, Family socioeconomic status, Friendship, Peer acceptance

## Abstract

**Background:**

Childhood loneliness is a significant public health concern, particularly in China due to distinct sociocultural contexts. Prior research often overlooks symptom-level interactions, limiting the precision of targeted interventions. This study applied network-based methodologies to clarify the structure of loneliness, identify intervention targets, and examine the roles of psychological factors and family socioeconomic status (SES).

**Methods:**

A total of 2,593 school-age children from Shantou, China, were assessed for loneliness, depressive symptoms, anxiety symptoms, ADHD symptoms, perceived social support, hope, and family SES. *In silico* interventions using the *NodeIdentifyR* algorithm (NIRA) within an Ising model identified effective targets for prevention and intervention. A Graphical Gaussian Model (GGM) mapped loneliness within a broader psychological context, and a Moderated Network Model (MNM) tested SES influences.

**Results:**

Lack of friendship and peer acceptance emerged as key targets for prevention and intervention, respectively. Loneliness functioned as both a central and bridging symptom in the psychological network, closely connected to other psychological variables. Higher SES buffered its associations with depressive symptoms and hope.

**Conclusions:**

Early, peer-focused, and context-sensitive strategies may more effectively support children’s well-being. This study is the first to apply network analysis and *in silico* intervention methods, providing novel perspectives and strategies for the prevention and intervention of childhood loneliness among Chinese children.

**Supplementary Information:**

The online version contains supplementary material available at 10.1186/s13034-025-00947-9.

## Introduction

Loneliness in childhood is increasingly recognized as a critical public health issue with lasting implications for children’s well-being and development [1]. Defined as a distressing emotional state arising from a mismatch between desired and actual social relationships [2], it affects approximately 20% of school-age children [3]. Beyond emotional and academic challenges [1], childhood loneliness is associated with long-term socioeconomic consequences, highlighting the need for early support [4]. Although loneliness interventions have largely focused on older adults, a recent systematic review found that only 2 of 22 rigorously evaluated trials targeted children, revealing a striking gap in child-focused research [5]. Moreover, it remains unclear which interventions work best for which children, as effectiveness varies by age, baseline loneliness, socioeconomic status, and intervention type [6]. These insights emphasize the necessity of targeted and context-sensitive interventions. In China, unique sociocultural factors including rapid socioeconomic transformation, rural–urban disparities, parental migration resulting in left-behind children, and growing academic pressure, add further complexity [7–9]. A deeper understanding of the core features, individual differences, and intervention opportunities surrounding childhood loneliness in China is therefore urgently needed.

Loneliness in childhood is a multifaceted emotional experience, closely intertwined with a range of other psychological factors. Growing evidence identifies loneliness as a transdiagnostic factor and a bridge linking core internalizing problems such as depression and anxiety [10, 11]. Children who feel persistently lonely often report elevated depressive and anxiety symptoms, as chronic social disconnection can lead to deep sadness and worry [12]. An umbrella review supports the longitudinal associations between loneliness, depressive symptoms, and suicide attempts [13], while network analysis highlights loneliness symptoms as key bridge nodes linking depression and social anxiety [14]. Loneliness is also associated with attention and behavioral difficulties: children with higher Attention-Deficit/Hyperactivity Disorder (ADHD) symptoms frequently experience greater loneliness, likely due to peer rejection and the social challenges commonly accompanying ADHD [15]. In contrast, positive social and cognitive resources appear to protect against loneliness. For instance, perceived social support—the sense of being cared for by family, peers, and others—is consistently associated with lower loneliness [16]. Similarly, hope, characterized by goal-directed thinking and perceived agency, is negatively related to loneliness, especially under adverse conditions [17]. From a systems perspective, individual differences in loneliness arise not only from the experience itself but also from its interactions with other psychological variables within person-specific psychological networks. Thus, understanding loneliness as embedded within such dynamic systems calls for a shift from examining isolated predictors toward modeling interdependencies among psychological variables, enabling a more comprehensive exploration of childhood loneliness.

Although children’s subjective experiences of loneliness have been examined, existing research largely relies on traditional latent factor models [18], overlooking the possibility that loneliness may arise from direct interactions among individual symptoms. In contrast, a network perspective conceptualizes symptoms as interconnected, mutually reinforcing components within a dynamic system [19]—an approach not yet applied to children’s loneliness. Moreover, investigations into psychological factors associated with loneliness typically use correlation-based analyses, which risk detecting spurious associations driven by shared covariance with unmeasured variables, thereby obscuring genuine interactions. As a result, conventional methods struggle to disentangle the complex relationships between loneliness and psychological factors, limiting the identification of independent contributors and obscuring underlying mechanisms. To date, few studies have adopted advanced network-based frameworks such as network analysis, *in silico* interventions, or moderated network models that explicitly capture these interdependencies and conditional influences at either the symptom level or across broader psychological domains [20].

In recent years, psychometric network analysis has become a powerful tool for exploring interactions among psychological variables. Unlike traditional models that focus on risk factors or impairments, the network perspective emphasizes how symptoms influence one another and collectively shape mental health [20]. By identifying central and bridge symptoms, network analysis can guide targeted interventions and clarify how children’s loneliness connects with related psychological variables, shedding light on potential causal mechanisms [21]. To support more precise clinical recommendations, researchers increasingly employ *in silico* interventions, such as those implemented by the *NodeIdentifyR* algorithm (NIRA) [22]. These simulations virtually amplify or reduce specific symptoms to estimate their impact on the broader network. Alleviating highly influential symptoms can reduce overall distress, while aggravating them may intensify it, making these symptoms key targets for intervention or prevention [23]. Applied to children’s loneliness, this approach offers an innovative, cost-effective way to test potential strategies before real-world implementation, enhancing clinical and educational precision. Moreover, understanding psychological symptoms within their social and cultural context is equally essential. Moderated Network Models (MNMs) address this by assessing how external factors influence relationships between symptoms. Using nodewise regression, MNMs detect how symptom interactions vary under different conditions [24]. Given the well-established impact of family socioeconomic status (SES) on Chinese children’s mental health [25], MNMs can reveal how SES may strengthen or weaken specific links between loneliness and related symptoms. In sum, integrating network analysis to map symptom interactions, NIRA to identify key intervention symptoms, and MNMs to account for contextual influences offers a deeper, culturally grounded understanding of children’s loneliness and psychological well-being in China.

In designing this study, we conceptualized loneliness at both the symptom and trait levels and adopted a dual strategy combining data-driven and hypothesis-driven approaches. At the symptom level, we applied psychometric network analysis and *in silico* interventions (NIRA) to examine interdependencies among loneliness symptoms and identify potential targets for intervention and prevention. At the trait level, grounded in established theoretical frameworks and prior empirical findings, we investigated children’s overall loneliness and its associations with depressive, anxiety, and ADHD symptoms, perceived social support, and hope, while incorporating family SES as a contextual moderator. This integrated approach was intended to balance conceptual rigor with openness to discovery, thereby advancing our understanding of childhood loneliness and informing targeted intervention strategies.

The present study aims to clarify the core features of loneliness among school-age children in China, identify effective targets for intervention and prevention, and explore psychological and socioeconomic contributors to individual differences in children’s loneliness. First, we constructed an item-level network of loneliness and applied the NIRA to perform symptom-specific *in silico* interventions, thereby identifying potential intervention targets. Second, recognizing that loneliness operates within a broader psychological context, we conducted domain- and facet-level network analyses to examine its associations with other psychological variables and to identify central and bridge symptoms across the psychological symptom network. Third, to account for contextual influences, we employed a MNM with family SES as a moderator, investigating how SES may influence associations between loneliness and related symptoms.

## Methods

### Participants

From March to June 2023, we conducted a cross-sectional survey using cluster random sampling. Students in grades 2 to 5 were randomly selected by class unit from four public elementary schools in Shantou, Guangdong Province, China. Grades 1 and 6 were excluded due to limited questionnaire suitability: first-graders were still adjusting to school routines, while sixth-graders were occupied with intensive preparation for middle school entrance exams. A total of 3,045 students took part, and 2,760 returned the questionnaires. We excluded those with missing loneliness data or over 30% missing responses items across all scales, yielding a final sample of 2,593 children (mean age = 10.18 ± 1.22 years; 51.3% boys).

Prior to data collection, teachers and research assistants received standardized training on the study’s objectives, procedures, and questionnaire content to ensure consistent survey administration. Teachers then distributed sealed envelopes containing written informed consent forms to students who expressed willingness to participate. These forms were taken home for review and signature by a parent or legal guardian. Only students who returned signed consent forms were invited to complete the self-report questionnaire. On the day of the survey, teachers and trained staff explained the questionnaire procedures using age-appropriate language and provided support as needed to ensure student comprehension and data quality. The study was approved by the Ethics Committee of Shantou University Medical College (SUMC-2023-016).

We calculated the needed sample size using the *powerly* R package with a Monte Carlo simulation [26], based on a 15-node cross-sectional network model. With a power of 0.80, network density of 0.40, and sensitivity of 0.60, the minimum recommended sample was 1,095, which was well below our final sample size.

### Measures

#### Children’s loneliness

The Children’s Loneliness Scale (CLS) was used to assess childhood loneliness [27]. The scale consists of 24 items: 16 primary items measuring feelings of loneliness, social adequacy, and peer status, and 8 supplementary items about hobbies help encourage honest responses. Children rated each item on a 5-point Likert scale, from “not true at all” to “always true.” Scores from the 16 primary items were summed, with 6 items (CLS01, CLS04, CLS08, CLS10, CLS16, and CLS22) reverse-scored to ensure that higher scores consistently reflected greater loneliness (e.g., a higher score on “have lots of friends” indicates fewer friendships). The CLS is widely used in China and demonstrated strong internal consistency in this study (Cronbach’s alpha = 0.832).

#### Children’s depressive symptoms

The Depression Self-rating Scale for Children (DSRS) was developed to evaluate depressive symptoms [28]. It includes 18 items rated on a 3-point scale: “never” (0), “sometimes” (1), and “most of the time” (2), with higher scores reflecting more severe symptoms. Two factors—low positive affect and negative affect—capture distinct dimensions of depressive symptoms [29]. The DSRS is suitable for use with Chinese children and exhibited acceptable reliability in this study (Cronbach’s alpha = 0.743).

#### Children’s anxiety symptoms

The Screen for Child Anxiety Related Emotional Disorders (SCARED) was used to assess anxiety symptoms in children [30]. This 41-item scale employs a 3-point rating system from 0 (“not true or hardly ever true”) to 2 (“true or often true”) and covers five dimensions: somatic/panic, general anxiety, separation anxiety, social phobia, and school phobia. The SCARED has been validated for use with Chinese children and showed excellent reliability in this study (Cronbach’s alpha = 0.918).

#### ADHD symptoms

The Abbreviated Symptom Questionnaire (ASQ), developed by Conners, was used to evaluate ADHD symptoms in children [31]. The ASQ includes 10 items, each rated on a 4-point scale from 0 (“never”) to 3 (“very frequent”), with the mean score used to calculate the hyperactivity index. A score of 1.5 or above suggests a potential risk for ADHD. The ASQ is widely used in China and is considered a reliable tool for both assessing ADHD and tracking treatment outcomes. In this study, it showed strong reliability (Cronbach’s alpha = 0.858).

#### Perceived social support

The Perceived Social Support Scale (PSSS) was used to investigate perceived social support among children [32]. The 12-item scale includes three subscales measuring support from Family, Friends, and a Significant Other. Children rated each item, with higher total scores indicating greater perceived support. In this study, the PSSS exhibited excellent internal consistency (Cronbach’s alpha = 0.932).

#### Children’s hope

The Children’s Hope Scale (CHS) is a six-item self-report tool that assesses hope in children [33], defined as a combination of agency and pathways thinking toward achieving goals. Responses are recorded on a 6-point scale, from 1 (“none of the time”) to 6 (“all of the time”), with higher scores indicating greater hope. The CHS has been validated for use with Chinese children and showed strong internal consistency in this study (Cronbach’s alpha = 0.842).

#### Family socioeconomic status

Family socioeconomic status (SES) was assessed using three key indicators: the highest education level and occupation of either parent, and household monthly income. Education was rated on a scale from 1 (primary school or below) to 5 (postgraduate education). Occupation was scored from 1 (unskilled workers and unemployed) to 5 (senior professionals and executive managers). Household monthly income was rated from 1 (less than 5,000 CNY) to 4 (more than 20,000 CNY). Each variable was standardized into a z-score, and a composite SES index was generated using principal component analysis (PCA) for further analysis.

### Statistical analyses

All analyses were conducted using R version 4.4.1. All tests were two-sided, with statistical significance set at *P* < 0.05.

#### Descriptive and preliminary analyses

Descriptive statistics were used to summarize sample characteristics, and Spearman correlation analysis was conducted to examine relationships between key variables. To identify redundant items in the psychometric network, we employed the topological overlap test using the *goldbricker* function from the *networktools* R package. This test compares item correlations to detect those measuring the same underlying construct (i.e., collinear items), with a significance threshold of *P* = 0.01 and a correlation threshold of 0.25 [34]. The analysis was conducted on the 16 primary items of the Children’s Loneliness Scale (CLS), excluding the 8 supplementary items. Item 20 was identified as highly redundant and was therefore removed, yielding a refined set of 15 core symptoms for subsequent network analyses. Details on data preprocessing—including the assessment of common method bias, handling of missing data, and covariate adjustments—are provided in the Supplementary Methods.

#### Recoding and dichotomization of the Children’s Loneliness Scale

Given that *in silico* interventions target individual nodes by increasing or decreasing their intercept terms (i.e., threshold parameters) in logistic regression relative to all other nodes, we employed the Ising model, which requires binary input [35, 36]. This binary modeling choice is theoretically grounded: social neuroscience conceptualizes loneliness as an adaptive “alarm signal” activated upon surpassing a critical threshold of social disconnection, akin to hunger or pain [37–39]. Psychometrically, many CLS items (e.g., “I feel alone”) naturally align with symptom presence or absence, and prior network psychometric research supports binary coding, demonstrating minimal information loss compared to continuous scales [40]. Binary loneliness indicators, such as those used in the UK Biobank, correlate strongly (*r* ≈ 0.88) with multi-item UCLA Loneliness Scale scores [41, 42]. Additionally, binary specification offers methodological clarity and interpretability in terms of conditional probabilities and facilitates intuitive, actionable simulations of symptom intervention.

The CLS items were originally rated on a five-point Likert scale; thus, we recoded them into binary variables to indicate the presence (1) or absence (0) of a loneliness symptom. Specifically, responses of “not true at all” (1) and “hardly ever true” (2) were coded as 0, while responses of “true sometimes” (3), “true most of the time” (4), and “always true” (5) were coded as 1. This dichotomization approach is consistent with our previous work using latent class analysis on children’s loneliness [43].

We recognize that simplifying Likert-scale data into binary form may affect the relationships among symptoms in the network. To evaluate this, we additionally fitted a Graphical Gaussian Model (GGM) using the original continuous CLS scores [44, 45]. We also validated the Ising network by calculating Spearman’s correlation coefficients to examine the strength of connections (edges) between nodes.

#### Network analyses and in silico interventions

Network models were estimated using the *estimateNetwork* function from the *bootnet* R package. The Ising model was fitted using *IsingFit* with binary input derived from 15 core loneliness symptoms [35], identified through the topological overlap test on the CLS. In parallel, two Graphical Gaussian Models (GGMs) were estimated *via EBICglasso* [44]. The first model included domain-level composite scores for children’s loneliness and related psychological variables: depressive symptoms, anxiety symptoms, ADHD symptoms, perceived social support, and hope. The second model was constructed at the facet level, incorporating 14 variables from the same domains to allow for a more granular analysis of interrelationships. Specifically, the facet-level GGM included: children’s loneliness (modeled as a single-factor construct); depressive symptoms (*Low positive affect*, *Negative affect*); anxiety symptoms (*Somatic/panic*, *General anxiety*, *Separation anxiety*, *Social phobia*, *School phobia*); ADHD symptoms (single-factor); perceived social support (*Family*, *Friends*, *Significant Other*); and children’s hope (*Agency*, *Pathways*). All facets were selected based on validated subscale structures of the respective instruments. Children’s loneliness and ADHD symptoms were retained as unified constructs due to their unidimensional nature in factor analytic assessments. The stability and accuracy of network parameters were assessed using two bootstrap methods implemented in *bootnet* [46]. To explore the moderating role of family SES, a Moderated Network Model (MNM) was applied using the *mgm* R package [24]. This model included domain-level scores for children’s loneliness and related psychological variables, with family SES specified as a continuous moderator. Parameter stability was tested with *mgm*’s *resample* bootstrap procedure. Centrality indices (strength, closeness, betweenness, expected influence) were computed using the *centralityPlot* function in *qgraph* [46], while bridge centrality metrics (bridge strength, bridge expected influence) were calculated using the *bridge* function from the *networktools* package [47]. *In silico* interventions were carried out using the *nodeIdentifyR* algorithm (NIRA) to identify symptom-specific intervention targets within the Ising model [22, 35]. A bootstrap resampling approach was used to evaluate the stability of intervention effects, with the mean and standard deviation of each node’s effect calculated to assess robustness and variability. All visualizations were created using the *qgraph* and *ggplot2* R packages. Additional details are available in the Supplementary Methods.

To further validate our findings, we conducted sensitivity analyses by adjusting for key covariates—including sex, grade, family SES, residence, and only-child status—in the estimation of GGMs. Each psychological variable was first regressed on these covariates using generalized linear models (Gaussian family). The residuals obtained were subsequently used as input for the domain- and facet-level GGM analyses. This approach ensured the associations observed in the network analyses reflect relationships independent of these background characteristics. For comparison, unadjusted GGMs are presented in the Supplementary Figures.

## Results

### Sample characteristics

A total of 2,593 students participated, with a mean age of 10.18 ± 1.22 years and an even gender split (51.3% boys). As shown in Table 1, students were evenly distributed across grades 2 to 5. Nearly half lived in rural areas (48.0%), and most had siblings (87.8%). Most parents had completed primary or secondary education, and about one-quarter had attended college or above. Mothers mainly worked in service, clerical, or manual jobs; fathers were more often in clerical or skilled manual roles. Over 70% of households reported a monthly income below 10,000 CNY.

**Table 1:** Demographic and socioeconomic characteristics of study participants (*N* = 2593)

Variable	*n* (%)/Mean (SD)
Sex	
Boys	1329 (51.3)
Girls	1264 (48.7)
**Age**	10.18 (1.22)
**Grade**	
Grade 2	613 (23.6)
Grade 3	640 (24.7)
Grade 4	693 (26.7)
Grade 5	647 (25.0)
**Ethnicity**	
Han Chinese	2554 (98.5)
Minority ethnic groups	28 (1.1)
Missing	11 (0.4)
**Place of residence**	
Rural	1244 (48.0)
Township	252 (9.7)
Urban	1086 (41.9)
Missing	11 (0.4)
**Only-child status**	
Only child	303 (11.7)
Non-only child	2276 (87.8)
Missing	14 (0.5)
**Family SES**	
*Maternal education*	
Primary school or below	610 (23.5)
Lower secondary education	940 (36.3)
Upper secondary education or vocational training	413 (15.9)
Tertiary education (associate or undergraduate)	605 (23.3)
Postgraduate education	16 (0.6)
Missing	9 (0.3)
*Paternal education*	
Primary school or below	316 (12.2)
Lower secondary education	1083 (41.8)
Upper secondary education or vocational training	492 (19.0)
Tertiary education (associate or undergraduate)	656 (25.3)
Postgraduate education	36 (1.4)
Missing	10 (0.4)
*Maternal occupation*	
Unskilled workers and unemployed	748 (28.8)
Skilled manual workers and self-employed	700 (27.0)
Clerical and service workers	845 (32.6)
Mid-level professionals and managers	114 (4.4)
Senior professionals and executive managers	171 (6.6)
Missing	15 (0.6)
*Paternal occupation*	
Unskilled workers and unemployed	429 (16.5)
Skilled manual workers and self-employed	699 (27.0)
Clerical and service workers	1057 (40.8)
Mid-level professionals and managers	223 (8.6)
Senior professionals and executive managers	168 (6.5)
Missing	17 (0.7)
*Household monthly income (CNY)*	
< 5000	661 (25.5)
5000~	1197 (46.2)
10,000~	530 (20.4)
> 20,000	188 (7.3)
Missing	17 (0.7)

Family SES = Family socioeconomic status. SD = Standard deviation. Household monthly income is reported in Chinese Yuan (CNY); 1 USD ≈ 7.3 CNY

### Ising network of children’s loneliness

**Fig. 1:**
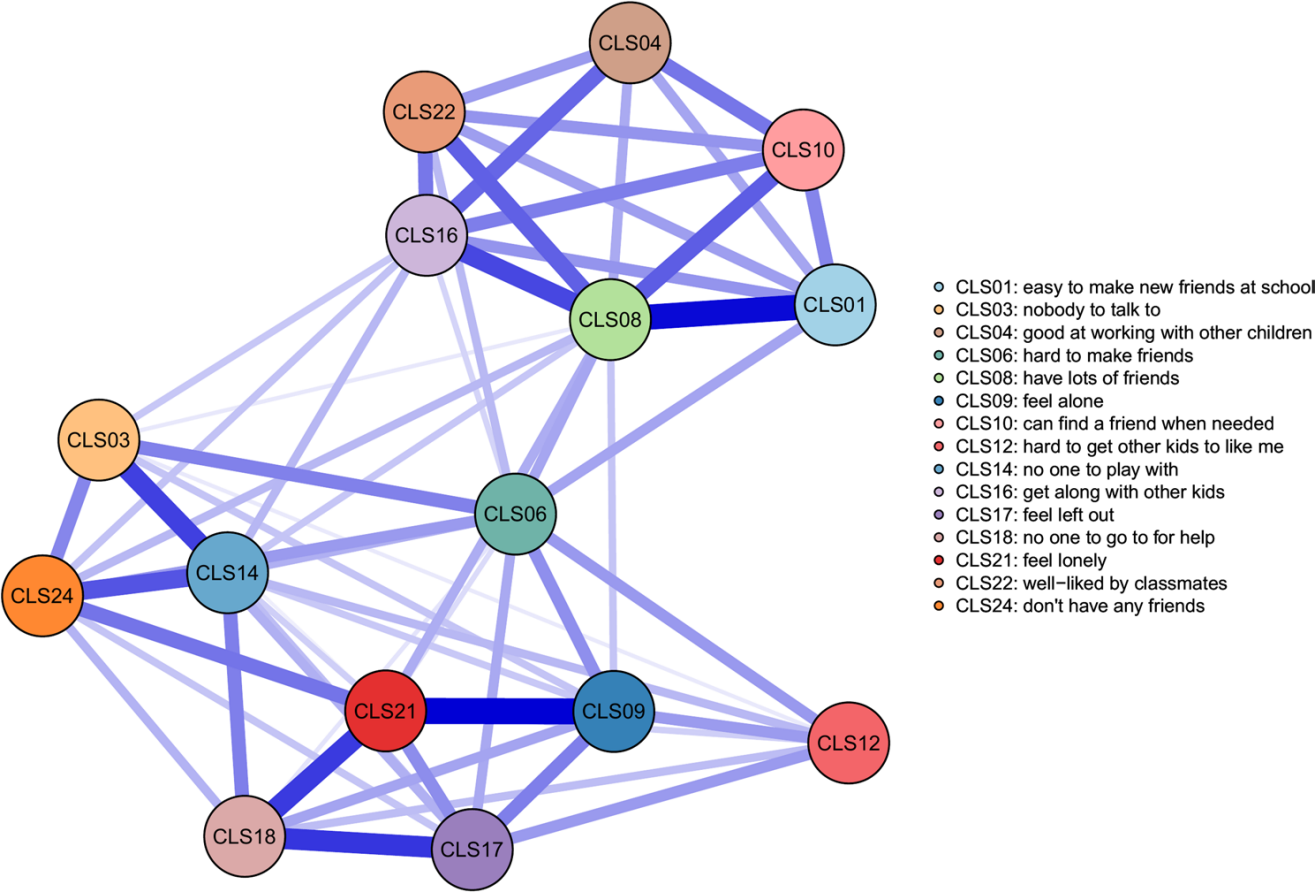
Ising network of loneliness symptoms in school-age children. *Notes*: Edges represent conditional dependencies between binary symptoms, estimated *via* logistic regression. Blue edges indicate positive associations; edge thickness reflects the magnitude of the regression coefficients

Table S1 presents descriptive statistics for children’s loneliness symptoms and the proportion of each symptom classified as active in the Ising network. As shown in Fig. S1, Spearman’s correlations indicated positive relationships among all variables. Figure 1 displays a dense Ising network of loneliness symptoms in school-age children, suggesting frequent symptom co-occurrence. CLS09 (“I feel alone”) was strongly connected with CLS21 (“I’m lonely”), and CLS01 (“easy to make new friends at school”) with CLS08 (“have lots of friends”), illustrating meaningful symptom clusters. Edge weights are detailed in Table S2. Centrality metrics (Table S1; Fig. S2) identified CLS08 (“have lots of friends”), CLS06 (“hard to make friends”), and CLS16 (“get along with other kids”) as the most central symptoms based on strength. Since the network contained no negative edges, expected influence was identical to strength and not reported separately. Accuracy and stability analyses (Fig. S3) showed edge weights were estimated with high precision, reflected by narrow confidence intervals. Centrality indices were stable, with strength (and expected influence) yielding stability coefficients above 0.5.

As the Ising model requires binary input, we dichotomized the CLS items for the main analysis. To assess the robustness of our findings, we additionally estimated a GGM using the original 5-point CLS scores (Fig. S4). The centrality results were largely unchanged: CLS08 still showed the highest strength, expected influence, and betweenness, while CLS06 remained highest in closeness. Full centrality results are shown in Fig. S5. Accuracy and stability analyses supported the reliability of the network (Fig. S6).

### Effects of *in silico* interventions

**Fig. 2:**
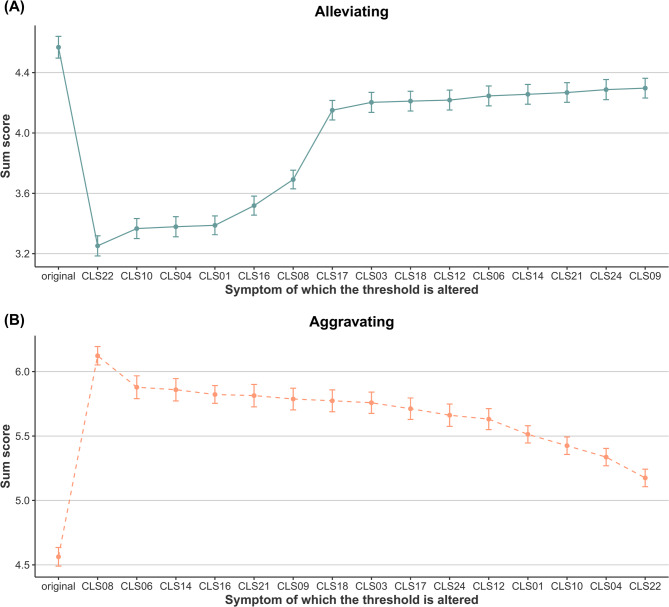
Simulated effects of alleviating (**A**) and aggravating (**B**) interventions on loneliness symptoms. *Notes*: Dots represent the network’s sum score, and lines indicate the 95% confidence interval. The x-axis shows symptoms whose thresholds were altered, including the original projected sum score based on simulations without intervention. Changes in sum scores reflect the projected effects of each symptom-specific intervention

We used NIRA to simulate interventions on each symptom in the Ising model and examined their projected effects. Alleviating CLS22 (“well-liked by classmates,” reverse-coded to indicate lower peer acceptance) significantly reduced overall symptom levels (Fig. 2A; Table S3), highlighting it as a promising target for clinical intervention. In contrast, simulating an increase in the item CLS08 (“have lots of friends,” also reverse-coded to indicate fewer friendships) markedly increased overall network symptom activation (Fig. 2B; Table S4). Given its high centrality, CLS08 may also serve as an effective target for preventing loneliness in school-age children.

We examined whether the predicted total symptom scores differed significantly after each simulated intervention. In the alleviation scenario, targeting all symptoms led to significantly lower loneliness scores compared to the original predictions, with the strongest effect for CLS22 (*t* = 26.279, *P* < 0.001; Table S3). In the aggravation scenario, scores increased significantly, particularly when CLS08 was targeted (*t* = −30.125, *P* < 0.001; Table S4).

To increase confidence in these findings, we conducted a bootstrap stability analysis with 5,000 iterations, each using 70% of the simulated participants sampled with replacement. As shown in Fig. S7, CLS22 and CLS08 exhibited the highest mean intervention effects under the alleviation and aggravation scenarios, respectively, with relatively low standard deviations across iterations. CLS22 consistently emerged as a promising treatment target, while CLS08 appeared to be key for prevention, further supporting the reliability of the NIRA results.

### Graphical Gaussian Model of loneliness and related psychological variables

**Fig. 3:**
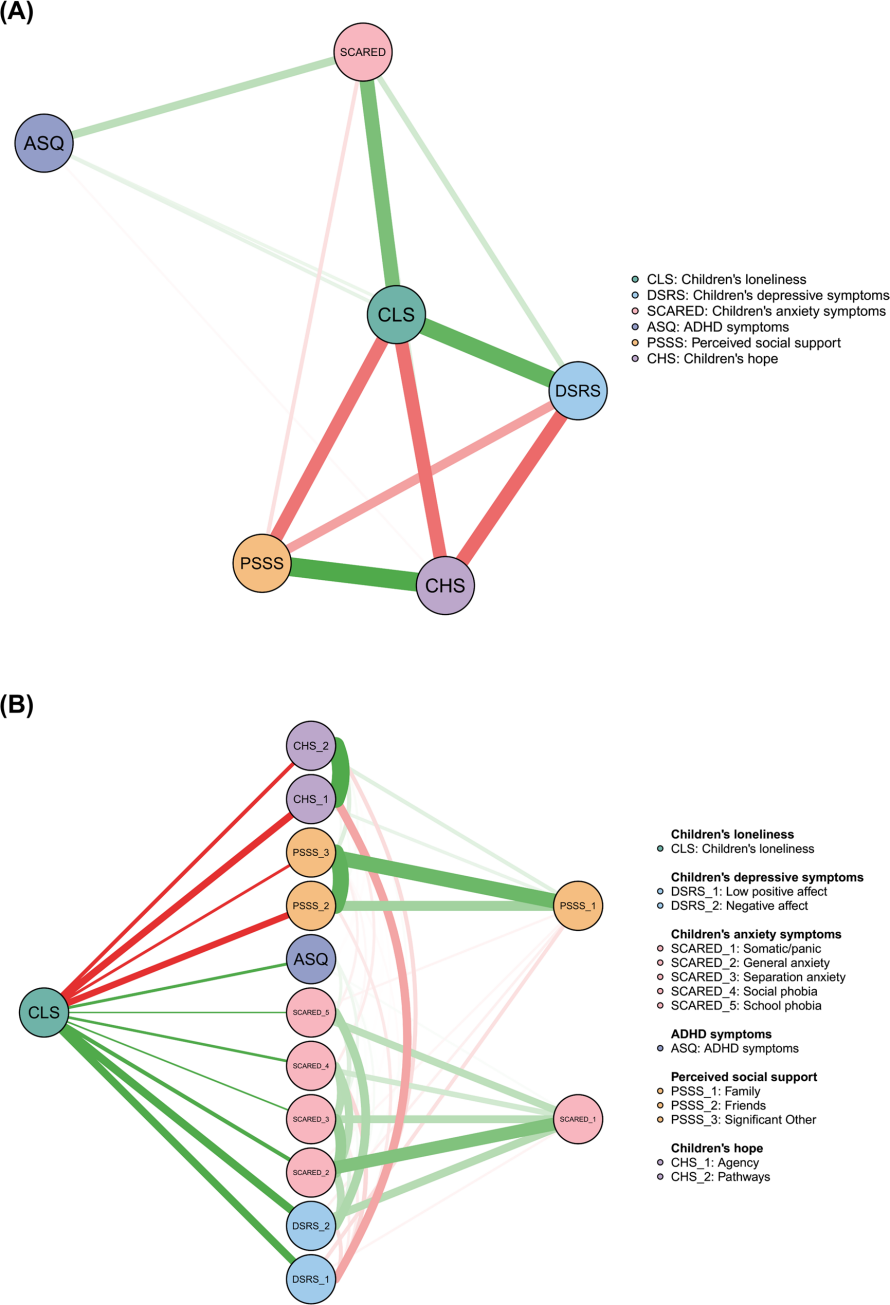
The relationships between children’s loneliness and related psychological variables in the domain-level (**A**) and facet-level (**B**) Graphical Gaussian Model (GGM). *Note*: Network analysis controlled for sex, grade, family SES, residence, and only-child status

Figure 3A presents the domain-level GGM of children’s loneliness and related psychological variables, with covariates controlled (see Table S5 for descriptive statistics; Fig. S8A for the model without covariates). Spearman’s correlations confirmed significant associations among all variables (Fig. S9A). In the network, perceived social support was strongly linked to children’s hope, while loneliness was closely connected to both depressive and anxiety symptoms (Table S6). To identify the most central variables, we focused on strength, closeness, and betweenness centrality, rather than expected influence, which accounts for the direction of associations. Across all three metrics, children’s loneliness ranked highest (Table S5; Fig. S10), indicating its central role in the psychological network. Accuracy and stability analyses (Fig. S11) supported the reliability of these results.

Given its central role in the domain-level network, loneliness was selected as the focal variable for constructing a facet-level flow network, shown in Fig. 3B (see Table S7 for descriptive statistics; Fig. S8B for the model without covariates). In this network, 11 symptoms were directly connected to children’s loneliness, and 2 were indirectly related. The strongest direct associations were with *negative affect* and *low positive affect*—both facets of depressive symptoms—followed by the *agency* component of hope (Table S8). Compared to the Spearman correlation matrix (Fig. S9B), the partial correlation network estimated using GGM was sparser (Table S8), with weaker associations reduced to zero. Some facets correlated with loneliness—such as *somatic/panic* anxiety and *family* support—did not show direct connections, suggesting their links to loneliness may be indirect and mediated by other psychological variables.

**Fig. 4:**
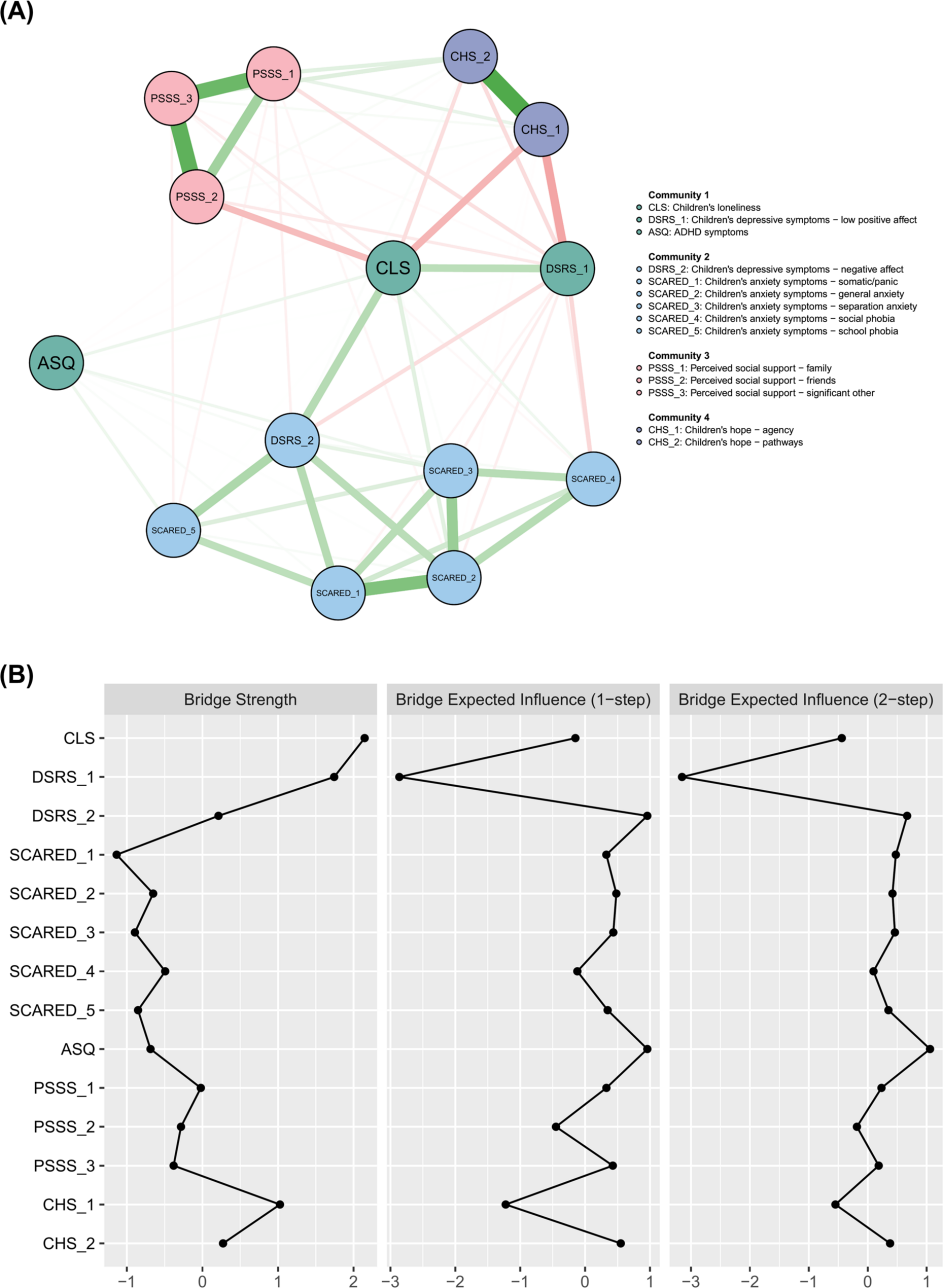
Bridge network analysis of loneliness and related psychological variables in the facet-level GGM. (**A**) The network shows communities detected *via* a spin-glass model with simulated annealing. (**B**) Bridge centrality metrics are presented as z-scores. *Note*: Bridge network analysis controlled for sex, grade, family SES, residence, and only-child status

To identify bridge symptoms that may contribute to comorbidity, we mapped the facet-level network using spin-glass community detection with simulated annealing (Fig. 4A). Loneliness showed the highest bridge strength, suggesting it may serve as a central link between co-occurring psychological symptoms. In contrast, ADHD symptoms had the highest bridge expected influence (EI1 and EI2; Fig. 4B). Despite fewer direct connections, their strong overall impact indicates a key role in spreading or triggering loneliness-related mental health issues. Accuracy and stability analyses (Fig. S12) confirmed the results’ reliability.

### Moderated Network Model with family SES as the moderator

**Fig. 5:**
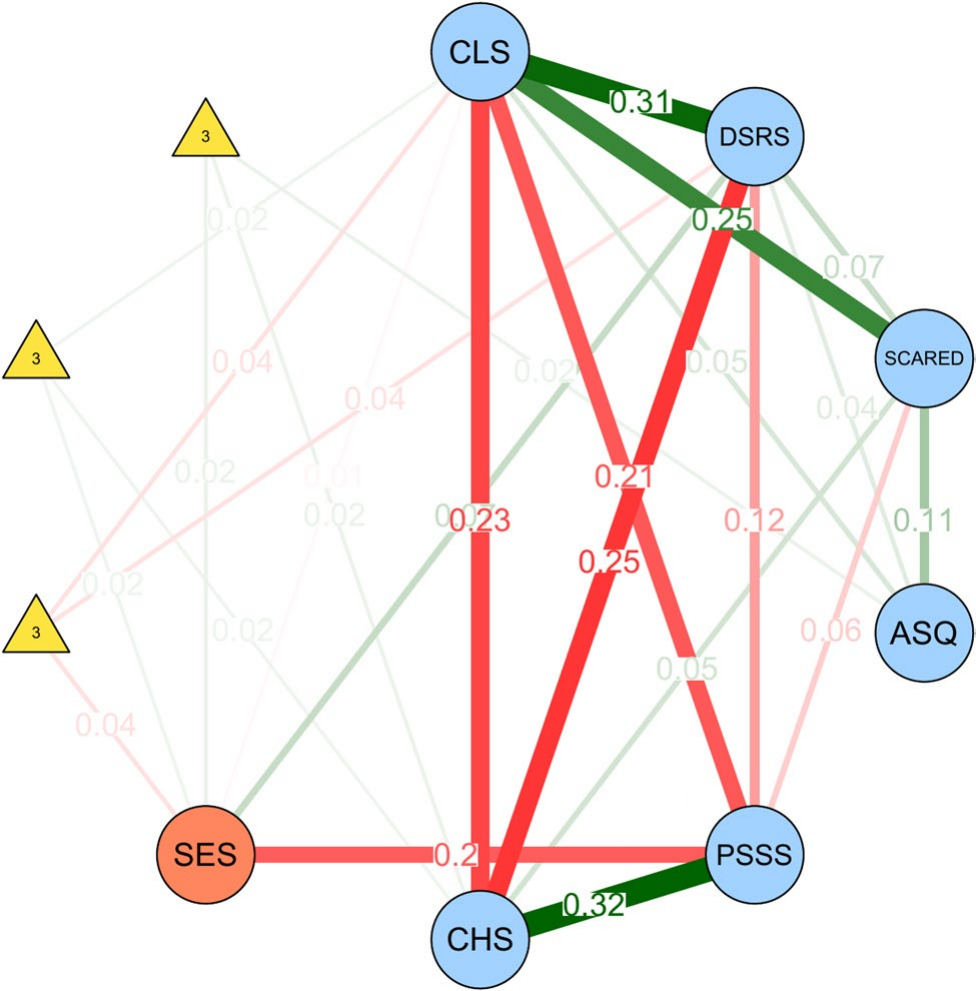
Moderated Network Model (MNM) with family SES as the moderator. *Notes*: Triangular nodes labeled ‘3’ indicate three-way interactions. Green edges indicate positive associations, while red edges indicate negative ones. Edge thickness and numeric labels represent the strength of pairwise or moderated effects. CLS = Children’s loneliness; DSRS = Children’s depressive symptoms; SCARED = Children’s anxiety symptoms; ASQ = ADHD symptoms; PSSS = Perceived social support; CHS = Children’s hope; SES = Family socioeconomic status

We identified three three-way interactions in the MNM with family SES as the moderator (Fig. 5). The first showed that SES moderated the relationship between children’s loneliness and depressive symptoms (weight = − 0.04). When SES was zero, the association was 0.31, decreasing by 0.04 with each one-unit increase in SES, suggesting a buffering effect. The second interaction showed that SES also moderated the association between loneliness and hope (weight = 0.02). This association was − 0.23 at SES = 0 and became less negative as SES increased, again indicating a protective role. The third interaction involved ADHD symptoms and hope (SES × ASQ × CHS, weight = 0.02). Although their direct association was weak, the interaction suggests their relationship may vary across SES levels. Bootstrapped estimates (Fig. S13, right panel) indicated that the SES effect on the loneliness–depressive symptoms link was the most stable (nonzero in 98.0% of bootstrap samples), with the other two slightly less robust but still supported.

### Summary of supplementary findings

Supplementary tables and figures provide detailed statistical outputs, including node activation frequencies (Table S1), network structures (Figs. S4, S8), edge weights (Tables S2, S6, S8), centrality and bridge metrics (Tables S1, S5, S7; Figs. S2, S5, S10), simulated intervention effects (Tables S3–S4), correlation structures (Figs. S1, S9), and model stability indices (Figs. S3, S6–S7, S11–S13). Specifically, Table S1 shows that CLS22 (“well-liked by classmates,” reverse-coded to indicate lower peer acceptance) is the most frequently activated symptom, while Table S2 details the strongest edges within the Ising network. Centrality and bridge metrics (Tables S1, S5, S7; Figs. S2, S5, S10) consistently identify CLS08 (“have lots of friends,” reverse-coded as lack of friendship) as the most central loneliness symptom and overall loneliness as a key hub within the psychological network, demonstrating robust stability (Figs. S3, S11–S12). Intervention simulations (Tables S3–S4) indicate alleviating deficits in peer acceptance (CLS22) reduces overall loneliness by approximately 1.32 points, whereas intensifying lack of friendship (CLS08) increases loneliness by about 1.56 points; both effects remain stable across 5,000 bootstraps (Fig. S7). Bootstrapped moderated models (Fig. S13) consistently retain significant SES × loneliness × depression and SES × loneliness × hope interactions in 98% and 88% of samples, respectively, further supporting the reliability of SES buffering effects. Together, these supplementary analyses reinforce the robustness and coherence of the network models, identifying key targets for intervention and prevention.

## Discussion

The present study employed network-based methodologies to characterize loneliness among Chinese school-age children, identify promising intervention and prevention targets, and examine how psychological factors and family SES shape individual differences in loneliness. Using NIRA within an Ising network, peer-related symptoms—specifically lack of friendship and peer acceptance—emerged as key targets for prevention and treatment, respectively. Domain- and facet-level GGM analyses further confirmed loneliness as a central component in the broader psychological network and revealed its bridging role, indicating loneliness may act as a crucial conduit for comorbid psychological conditions. The MNM demonstrated that family SES buffered associations between loneliness and both depressive symptoms and hope. These findings advance our understanding of childhood loneliness and individual variability, suggesting potential strategies for addressing it early in life.

### Fostering friendship and peer acceptance: core strategies for loneliness prevention and intervention

Lack of friendship (reflected by reverse-coded responses to “have lots of friends”) was identified as both a central symptom and the most sensitive intervention target in simulations. This finding reinforces the conceptual coherence that a lack of friendships significantly exacerbates childhood loneliness, thereby propagating distress throughout the symptom network. Consistent with prior NIRA research [48], the symptom with the highest centrality also showed the greatest response to aggravating interventions, reinforcing its preventive significance. Lodder et al. (2017) found that adolescents with fewer reciprocated friendships reported higher loneliness than those with many friends [49]​. A classic study similarly showed that even unpopular children feel less lonely if they have a best friend [50]. From a self-determination theory perspective, this is intuitive—friendships meet the basic need for relatedness by providing companionship, validation, and support. When this need goes unmet, loneliness and isolation often follow. Therefore, bolstering friendships is a promising preventive strategy, helping buffer against loneliness by fulfilling children’s need to belong. Schools can promote peer connection through structured approaches such as buddy systems or peer-partner programs, ensuring each child—especially those who are shy or new—has a designated peer. Thoughtful pairing based on shared interests or complementary traits can foster natural rapport and social inclusion. Incorporating cooperative learning tasks into daily classroom routines can further strengthen peer bonds and build social self-efficacy, helping children develop confidence in their ability to form friendships and be accepted by peers.

Peer acceptance (“well-liked by classmates”) emerged as a key protective factor against loneliness. Simulated increases in peer acceptance significantly reduced loneliness symptoms, suggesting that feeling accepted directly alleviates loneliness. Antonopoulou et al. (2019) demonstrated that peer acceptance moderately protected primary school children from loneliness [51]. Being well-liked not only offers more opportunities for companionship but also enhances self-worth through a sense of belonging. According to need-to-belong theory, humans fundamentally seek positive relationships and acceptance; failure to meet this need leads to loneliness and distress. Unsurprisingly, children’s perception of being liked is closely tied to their loneliness levels. Supporting this, research in rural China found that peer acceptance buffered the impact of emotional abuse on loneliness among left-behind adolescents, with gender differences observed among non-left-behind youth [52]. Thus, the protective role of peer acceptance highlights its relevance as an intervention focus—further supported by our alleviating intervention results. School-based efforts to promote peer acceptance offer a promising pathway to reduce loneliness and enhance social integration. Teachers play a pivotal role by modeling warmth and inclusion, particularly toward socially isolated students. Small but visible actions, such as engaging warmly with withdrawn students, assigning meaningful roles, or highlighting strengths, can shift peer perceptions and elevate a student’s social standing. Class-wide activities that celebrate each child’s contributions, such as regular “kindness sessions” or empathy-focused storytelling, help cultivate a culture of mutual respect and belonging. Incorporating social-emotional learning (SEL) into the curriculum further develops essential friendship skills, including empathy, active listening, and conflict resolution [53]. Through role-playing and guided practice, students learn to cooperate, communicate, and navigate conflict, laying the foundation for positive and lasting peer relationships.

Notably, the two key intervention targets—“have lots of friends” and “well-liked by classmates”—are positively worded items. Children reporting high loneliness often could not endorse these statements, suggesting a lack of confidence in recognizing their social strengths. This aligns with our earlier findings among Chinese school-age children, where a “borderline loneliness group” (about 40.4% of the sample) showed low confidence in positively framed social competence measures [43]. Similarly, lonely children often rated themselves lower in scholastic and social competence and had poorer self-worth [54]. They tend to expect rejection and doubt their ability to make friends, leading to withdrawal or anxious behaviors that further limit peer engagement—reinforcing a cycle of loneliness. These insights highlight the importance of not only creating inclusive peer environments but also strengthening children’s self-efficacy. Confident children are more likely to approach peers and display positive behaviors, which fosters better peer responses and friendships, ultimately boosting self-esteem.

Careful interpretation is necessary due to Chinese cultural norms emphasizing collectivism and group harmony, which may influence school-age children’s responses to positively worded or peer-related self-report items. In Chinese culture, humility and self-effacement are valued for maintaining social cohesion, thus leading children to downplay personal successes or popularity to adhere to modesty norms [55, 56]. Consequently, this social desirability bias often results in Chinese children assigning themselves lower ratings on self-competence and positive traits compared to their Western peers. For instance, research indicates Chinese children perceive a “modest lie” (understating strengths) more favorably than an “immodest truth” (acknowledging accomplishments openly) [56]. Thus, culturally driven modesty may lead children to under-endorse positively worded statements like “I have lots of friends” from measures such as the Children’s Loneliness Scale (CLS). Such statements requiring selfaffirmation might feel culturally uncomfortable, leading children to provide neutral or negative responses, thereby artificially inflating measured loneliness. Notably, recent shifts toward individualism in urban Chinese society have seen newer cohorts reporting lower loneliness than earlier cohorts from the 1990 s [57], suggesting evolving cultural norms may gradually mitigate these biases. Therefore, recognizing the influence of modesty norms and social desirability biases is essential for accurately interpreting CLS data within Chinese collectivist contexts.

### Loneliness as a key conduit: the central and bridging role and its psychological correlates

In our GGMs, loneliness was positively associated with psychopathological symptoms—including depression, anxiety, and ADHD—and negatively linked to psychosocial resources like hope and perceived social support. Its strongest connections were with depressive symptoms, and the most pronounced negative edge was with hope, particularly agency. This mirrors prior evidence that loneliness is closely tied to internalizing distress [58], while hope functions as a protective factor [59].

Loneliness emerged as the most central node across strength, betweenness, and closeness metrics, indicating it not only connects with many symptoms but also occupies a structurally influential position within the network. In psychological terms, loneliness’s centrality suggests that it may not only co-occur with various symptoms but also serve as an integrative hub that amplifies or perpetuates symptoms, facilitating cycles of distress. This supports prior studies identifying loneliness as central in depressive networks of school-age children and adolescents, respectively [40, 60]. Our results extend this by demonstrating loneliness’s centrality across a broader child mental health network—including depressive and anxiety symptoms, ADHD symptoms, hope, and perceived social support—implying that loneliness may serve as a common thread linking diverse emotional and behavioral problems in childhood. However, loneliness appears less central in adult networks [61], pointing to developmental and contextual factors shaping its role.

Beyond centrality, bridge metrics provide additional insight into nodes that connect symptom clusters, potentially serving as gateways for co-occurring symptoms. Loneliness showed the highest bridge strength, positioning it as a key gateway and reinforcing its role as a transdiagnostic intervention target. This aligns with evidence indicating loneliness as a general risk factor for mental health problems [62]. Youth network studies have similarly highlighted specific loneliness experiences—like feeling that “People are around me but not with me”—as critical bridges connecting depression and anxiety [58]. Thus, interventions targeting loneliness might effectively disrupt or prevent cascading effects across symptom clusters.

ADHD symptoms appeared relatively isolated, with few strong direct connections, yet exhibited the highest bridge expected influence. This suggests that, despite their peripheral position, ADHD symptoms may function as hidden “amplifiers” or “gatekeepers,” subtly channeling behavioral dysregulation into emotional distress. This supports prior findings that hyperactivity/impulsivity symptoms may bridge ADHD and internalizing problems [63]. Clinically, addressing these bridging ADHD symptoms may help interrupt the progression of emotional and behavioral problems, preventing broader comorbidities. In sum, both loneliness and ADHD act as essential network bridges, but in distinct ways: loneliness connects broadly to psychosocial factors, while ADHD, though peripheral, uniquely bridges externalizing behaviors to the wider network.

Understanding these bridging roles is vital for developing targeted interventions aimed at disrupting vicious cycles and reducing overall psychological distress. On one hand, closing the loneliness gateway through school-based social skills training, structured peer mentoring, and cognitive behavioral therapy can weaken loneliness’s hub and gateway functions by reducing social skill deficits and cognitive distortions [64], reducing the risk of self-reinforcing internalizing cycles. On the other hand, regulating the ADHD gateway with executive function coaching, mindfulness-based self-regulation [65], and, when appropriate, pharmacotherapy may help prevent the spread of comorbid symptoms across behavioral and emotional domains.

### Family SES as a protective moderator: buffering the links between loneliness, depressive symptoms, and hope

Our findings suggest that higher family SES buffers the relationship between loneliness and depression, consistent with previous studies. For instance, loneliness had a stronger depressive impact on disadvantaged “left-behind” youth in rural China [66], and a cross-national study found higher SES reduced vulnerability to loneliness-related depression in older adults [67]. These studies collectively suggest low SES amplifies the mental health burden of loneliness, while greater family resources potentially offer protective mechanisms, including effective parenting practices, enriched environments, and access to mental health services. One significant buffering mechanism involves parenting styles. Higher-SES parents typically have greater educational and financial resources, thus facilitating more supportive parenting practices and a nurturing home environment. A large longitudinal study in Australia identified negative parenting style as the predominant factor contributing to mental health disparities related to SES, explaining over half of the differences observed [68]. Another key mechanism is access to enriched home and school environments. Empirical evidence demonstrates that higher-SES parents actively support their children’s participation in structured activities and hobbies, enhancing peer engagement and reducing isolation [69]. Furthermore, children from high-SES families often live in safer neighborhoods and attend schools with more supportive climates, alleviating chronic stressors linked to loneliness. Additionally, greater SES provides enhanced access to mental health care and reduces stigma around mental health service use [70]. Economically advantaged families can more readily access high-quality mental health services, such as counseling or therapy. Early and open engagement with mental health resources can disrupt the progression from loneliness to depression by teaching effective coping strategies and facilitating social reconnection. However, it is important to acknowledge divergent findings. For instance, one study of Chinese school-age children reported no significant association between SES and psychological well-being [71], suggesting variability based on contextual factors.

We also found that higher family SES weakens the negative association between loneliness and hope. Families with greater resources often provide emotional support, security, and opportunities that foster a more hopeful outlook in children. High-SES parents invest more in their children’s development by leveraging financial, social, and human capital. This investment often manifests as greater parental involvement and responsiveness, bolstering a child’s sense of security and hope. Reduced stigma in high-SES communities contributes to a more positive outlook on emotional well-being. Evidence suggests that youth from wealthier or more educated families report higher levels of goal-directed hope and optimism than their lower-SES peers [72]. Conversely, socioeconomic adversity is linked with a more negative or uncertain outlook on the future. A hopeful child is less likely to interpret loneliness as permanent, viewing it as a temporary and surmountable challenge. High-SES contexts nurture optimism through exposure to successful role models, reassurance about future opportunities, and encouragement of problem solving. Overall, psychological resources such as hope and self-efficacy, more abundant in higher SES environments, help counter negative cognitions associated with chronic loneliness. This protective role mirrors SES’s buffering effect on depression: just as wealth and education can cushion loneliness’s emotional toll, they can also preserve hope in the face of social isolation. Although research on SES as a moderator of the loneliness–hope relationship in youth is limited, evidence suggests the necessity of both economic and psychological support to sustain hope in lonely youth [73].

In conclusion, context matters in how loneliness affects children. Higher family SES offers a protective buffer, reducing its effects on depression and hope, though not fully eliminating them. Variability across studies suggests SES interacts with broader developmental factors. Interventions should focus on enhancing supportive relationships and personal resilience, especially in low-SES environments where protective resources may be scarce.

### Limitations

This study has several limitations. First, its cross-sectional design limits causal inferences; longitudinal research is needed to examine temporal dynamics. Second, our sample was drawn solely from southern China, which may restrict generalizability. Although the large sample enhances statistical power, the findings may not extend to regions with different sociocultural, linguistic, or educational contexts. Replication in other parts of China and in culturally diverse populations—especially in Western settings where loneliness may manifest differently—is necessary to establish broader applicability. Third, while the *NodeIdentifyR* algorithm facilitates virtual interventions simulations, their clinical effectiveness requires further validation. Finally, measurement constraints may have biased loneliness estimates: Chinese collectivist modesty norms may have led children to under-endorse positively worded Children’s Loneliness Scale items, while dichotomizing the five-point scale likely reduced sensitivity to severity and obscured nuanced symptom relationships in the network.

## Conclusion

In summary, this study offers a network-based exploration of childhood loneliness among school-age children in China. *In silico* interventions suggest friendship and peer acceptance as potential targets for prevention and treatment. Loneliness was identified as a key symptom, functioning both centrally and as a bridge between psychological conditions, indicating its significant role within symptom networks. Additionally, moderated network analysis revealed that higher family socioeconomic status may buffer the association between loneliness and depressive symptoms, as well as between loneliness and hope, thus highlighting the potential moderating influence of contextual factors. These findings point to the importance of early, peer-focused, and context-sensitive interventions. Educational institutions and families might benefit from organizing group activities and promoting social skills training to mitigate childhood loneliness by improving the quality of children’s friendships and peer acceptance. Future longitudinal research is necessary to establish causality and further refine intervention approaches to support children’s mental health effectively.

## Electronic supplementary material

Supplementary Material 1

## Data Availability

The data used and/or analyzed during the current study are available from the corresponding author on reasonable request.

## Abbreviations

NIRA: *NodeIdentifyR* algorithm
GGM: Graphical Gaussian Model
MNM: Moderated Network Model
SES: Socioeconomic status
ADHD: Attention-Deficit/Hyperactivity Disorder
CLS: Children’s Loneliness Scale
DSRS: Depression Self-rating Scale for Children
SCARED: Screen for Child Anxiety Related Emotional Disorders
ASQ: Abbreviated Symptom Questionnaire
PSSS: Perceived Social Support Scale
CHS: Children’s Hope Scale
