# Supplementary material for: Targeting childhood loneliness in china: in silico interventions and moderated network analysis
Source: Child Adolesc Psychiatry Ment Health. 2025 Jul 26;19:89. doi: 10.1186/s13034-025-00947-9 (PMC12297741; doi:10.1186/s13034-025-00947-9)
Supplement: Supplementary file 1 — Supplementary Material 1 [file 13034_2025_947_MOESM1_ESM.docx]

**Targeting childhood loneliness in China:**

***In silico* interventions and moderated network analysis**

Xinle Yu^1,2^, Xuanzhi Zhang^1,2^, Kusheng Wu^3^, Zhenqiang Xu^1,2^, Zhiya Liang^4^, Wanyi Wen^5^, Dinghui Wang^1,3^, Yanhong Huang^1,2,*^

^1^ Mental Health Center of Shantou University, Shantou, Guangdong, China

^2^ Shantou University Medical College—Faculty of Medicine of University of Manitoba Joint Laboratory of Biological Psychiatry, Shantou, Guangdong, China

^3^ Department of Preventive Medicine, Shantou University Medical College, Shantou, Guangdong, China

^4^ School of Public Health, Zhaoqing Medical College, Zhaoqing, Guangdong, China

^5^ The People’s Hospital of Gaoming District of Foshan City, Foshan, Guangdong, China

^*^Corresponding author:

Yanhong Huang, Mental Health Center of Shantou University, North Taishan Road, Shantou 515065, China. Email: 12yhhuang@stu.edu.cn

**Supplementary Materials**

***Supplementary Methods***

Data processing and analyses

***Supplementary Tables***

Table S1 Descriptive statistics and centrality metrics of Children’s Loneliness Scale (CLS) items

Table S2 Edge weight matrix of the Ising network for children’s loneliness symptoms

Table S3 Simulated changes in total loneliness scores in response to alleviating interventions at each node

Table S4 Simulated changes in total loneliness scores in response to aggravating interventions at each node

Table S5 Descriptive statistics and centrality metrics of loneliness and related psychological variables in the domain-level Graphical Gaussian Model (GGM)

Table S6 Edge weight matrix of loneliness and related psychological variables in the domain-level GGM

Table S7 Descriptive statistics and bridge centrality metrics of loneliness and related psychological variables in the facet-level GGM

Table S8 Edge weight matrix of loneliness and related psychological variables in the facet-level GGM

***Supplementary Figures***

Fig. S1. Spearman’s correlation matrix of children’s loneliness symptoms

Fig. S2. Centrality metrics of children’s loneliness symptoms in the Ising network (presented as z-scores)

Fig. S3. Stability and accuracy analysis of the Ising network

Fig. S4. GGM of loneliness symptoms in school-age children

Fig. S5. Centrality metrics of children’s loneliness symptoms in the GGM (presented as z-scores)

Fig. S6. Stability and accuracy analysis of the GGM

Fig. S7. Bootstrap analysis of key node stability under alleviating and aggravating interventions

Fig. S8. The relationships between children’s loneliness and related psychological variables in the domain-level and facet-level GGM (no covariates controlled)

Fig. S9. Spearman’s correlation matrix of children’s loneliness and related psychological variables in the domain-level and facet-level GGM

Fig. S10. Centrality metrics of children’s loneliness and related psychological variables in the domain-level GGM (presented as z-scores)

Fig. S11. Stability and accuracy of the domain-level GGM for loneliness and related psychological variables

Fig. S12. Stability and accuracy of the facet-level GGM for loneliness and related psychological variables

Fig. S13. Bootstrapped sampling distributions in the Moderated Network Model

**Supplementary Methods: Data Processing and Analyses**

We conducted data analyses in R 4.4.1 within RStudio, using the *bootnet*, *networktools*, *nodeIdentifyR*, *mgm*, *qgraph*, *ggplot2*, *igraph*, and *dplyr* R packages.

1. **Preliminary analyses**
   1. *Common method bias*

Harman’s one-factor test was applied to detect the common method variance [1]. The analysis indicated that 18 factors had eigenvalues greater than 1, and the first factor explained 14.87% of the total variance—well below the 40% threshold. This suggests that common method bias is unlikely to be a major issue in this study.

- 1. *Missing data handling*

No missing values were found in the loneliness data, and missing values in other variables were imputed using Multivariate Imputation by Chained Equations (MICE) through the *mice* R package [2].

- 1. *Covariate adjustment for network estimation*

In the GGM analysis of loneliness and psychological variables at both the domain and facet levels, we controlled for key covariates—sex, grade, family socioeconomic status (SES), residence, and only-child status. Each variable was first regressed on these covariates using generalized linear models (Gaussian family), and the resulting residuals were then used for network estimation [3]. This approach helps ensure that the associations observed in the network reflect relationships beyond the influence of these background characteristics.

1. **Network analyses**
   1. *Ising network & Graphical Gaussian Model*
      1. *Network estimation*

We estimated network models using the *bootnet* R package [4]. To examine children’s loneliness symptoms and prepare for future *in silico* interventions, we applied the Ising model with the *IsingFit* method, which uses LASSO-regularized logistic regression to identify conditional dependencies among binary symptoms [5]. For broader relationships between loneliness and psychological variables, we used the GGM with the *EBICglasso* method, which applies graphical LASSO along with Extended Bayesian Information Criterion (EBIC) model selection to estimate partial correlations among continuous variables [6, 7].

We used the *qgraph* R package to draw the networks using a “spring” layout [8], where symptoms are shown as nodes and their connections as edges. Blue edges (in the Ising network) and green edges (in the GGM) show positive links between symptoms, while red edges show negative ones. The thicker the edge, the stronger the connection.

- - 1. *Network inference*

We used the *qgraph* R package to calculate centrality metrics and identify key symptoms in the network [8]. Specifically, we computed node strength, closeness, betweenness, and expected influence using *qgraph*’s *centrality* function [9, 10]. Strength reflects how strongly a symptom is directly connected to others, based on the total absolute weight of its links. Closeness indicates how quickly a symptom can reach all others in the network. Betweenness shows how often a symptom lies on the shortest paths between other symptoms, highlighting its role as a bridge or connector. We also calculated expected influence, which sums the signed edge weights connected to a node, capturing a symptom’s overall impact while accounting for both positive and negative associations. All centrality values were standardized using z-scores to allow for direct comparison. These metrics help identify the most central symptoms, which may serve as promising targets for intervention due to their strong and widespread influence within the network.

To assess bridge centrality in the facet-level GGM of loneliness and psychological variables, we applied the *bridge* function from the *networktools* R package to compute key metrics including bridge strength, which sums the absolute weights of edges connecting a node to other communities, and bridge expected influence (bridge EI), which sums the signed edge weights to reflect whether those connections are positive or negative [11]. To further assess how nodes transmit activation across communities, we calculated two-step bridge expected influence. This metric captures a node’s impact on other communities both directly (bridge EI1—the total strength of its direct links to nodes in other clusters) and indirectly (bridge EI2—the influence it exerts through its neighboring nodes). Higher values indicate greater cross-community influence. Symptom communities were identified using the *cluster_spinglass* function from the *igraph* R package. These metrics allowed us to identify bridge symptoms—nodes that connect different symptom clusters and may contribute to comorbidity or co-activation [12]. Targeting these symptoms may help disrupt harmful connections between symptom groups.

- - 1. *Network accuracy and stability*

To estimate the accuracy of network edge weights, we used a non-parametric bootstrap method with 1,000 resamples to calculate 95% confidence intervals (CIs) for each edge [4]. In the graph, red dots represent the original edge weights, while black dots show the bootstrap estimates, ordered from highest to lowest. The gray shaded area indicates the 95% CIs. Narrower intervals suggest greater precision in the edge weight estimates.

To evaluate the stability of both centrality and bridge centrality metrics, we conducted a centrality stability analysis using the central stability coefficient (CS coefficient) [4]. This coefficient indicates the proportion of data that can be removed while still maintaining, with 95% confidence, a strong rank correlation (r ≥ 0.7) between the original and subset-based centrality values. A CS coefficient above 0.5 suggests strong stability, values between 0.25 and 0.5 indicate moderate stability, and values below 0.25 reflect low stability.

- 1. *Moderated Network Model*

We applied a Moderated Network Model (MNM) using the *mgm* R package to explore how family SES influences the relationships among children’s loneliness, depressive symptoms, anxiety symptoms, ADHD symptoms, perceived social support, and hope [13]. Family SES was treated as a continuous moderator to examine its impact on these associations.

The MNM estimates both pairwise and three-way interactions using nodewise LASSO regressions, which help identify direct relationships between variables while accounting for moderation effects [14]. All variables were assumed to follow a Gaussian distribution, and the LASSO penalty parameter (λ) was selected via cross-validation [15]. While this method improves sensitivity in detecting true relationships, it may also introduce false positives. To reduce this risk, we applied the conservative AND rule, which retains only those edges that consistently appeared across all nodewise regressions. This increases the specificity of the model compared to the OR rule, which includes edges if they appear in at least one regression.

For visualization, we used the *qgraph* R package with the “circle” layout. Nodes represent variables, and edges represent conditional associations after controlling for all others. Green edges indicate positive associations, red edges indicate negative ones, and edge thickness reflects the strength of the connection.

To assess the stability of the network, we conducted a nonparametric bootstrap analysis with 1,000 resamples [13, 14]. We used the sampling distributions of the estimated interactions to calculate mean coefficients and 95% confidence intervals (CIs). Edges with CIs that did not include zero were considered stable and likely to reflect meaningful associations rather than random noise.

1. ***In silico* interventions**

To identify the most effective symptom-specific intervention targets within the network model, we conducted *in silico* interventions using the *nodeIdentifyR* algorithm (NIRA) [16]. This simulation-based method, based on the Ising model, systematically alters individual symptoms to estimate their projected impact on overall symptom activation across the network [17].

- 1. *Ising network estimation*

We estimated the Ising network using logistic regression, where each symptom was iteratively regressed on all other symptoms in the network [5]. The regression coefficients (edge weights) represent the strength and direction of connections between symptoms, while the intercept (threshold parameter) reflects how likely each symptom is to activate on its own.

- 1. *Simulation-based interventions*

Simulations were performed using the *IsingSampler* R package, which employs the Metropolis-Hastings algorithm to generate samples from the Ising model. We generated 5,000 simulated “participants” for each condition. First, a baseline simulation was run using the original thresholds. Then, for each symptom, two types of simulated interventions were administered: alleviating interventions (lowering the symptom’s threshold) and aggravating interventions (raising the symptom’s threshold). The magnitude of each intervention was set to twice the standard deviation of all symptom thresholds, ensuring comparability across symptoms [16, 18]. In total, we conducted 16 simulations (1 baseline + 15 symptom-specific interventions), resulting in 16 × 5000 simulated observations. By comparing overall symptom activation levels between the baseline and each intervention, we quantified which symptoms had the greatest impact on the network, thereby identifying the most effective symptom-specific targets [19].

- 1. *Evaluation of intervention efficacy*

We assessed the effect of each intervention on loneliness levels using multiple independent *t*-tests, comparing each intervention condition to the baseline [20]. To account for multiple comparisons, the Bonferroni correction was applied to reduce the chance of false positives.

- 1. *Bootstrap stability analysis*

To test the robustness of our intervention findings, we conducted a bootstrap stability analysis. In each of 5,000 iterations, we randomly resampled 70% of the simulated participants with replacement. For each iteration, we calculated the mean intervention effect for each symptom and tracked the variability of these effects across iterations, measured by standard deviation. This allowed us to assess how consistently each symptom responded to the intervention across different subsamples of individuals. The analysis helped identify the most stable and effective intervention targets, increasing confidence in the NIRA results.

- 1. *Reproducibility and code availability*

To enhance transparency and facilitate replication of our *in silico* intervention analysis using NIRA, we provide the complete R code used in our study below.

*# Load the dataset*

data <- read.csv(‘data.csv’, header = TRUE)

*# Fit the Ising model*

library(bootnet)

gs_fit<-estimateNetwork(data, default = “IsingFit”)

*# Run NIRA-based in silico interventions*

library(nodeIdentifyR)

library(dplyr)

edgeWeightMatrix<-gs_fit$graph

thresholdVector<-gs_fit$intercepts

*# Simulate “alleviating” interventions (alternative: “aggravating”)*

gs_IsingSamples<-simulateResponses(edgeWeightMatrix, thresholdVector, “alleviating”, 2)

*# Calculate and visualize symptom activation*

gs_sumIsingSamples<-calculateSumScores(gs_IsingSamples)

gs_sumIsingSamplesLong<-prepareDFforPlottingAndANOVA(gs_sumIsingSamples)

plotSumScores(sum_scores_long=gs_sumIsingSamplesLong,perturbation_type=“alleviating”)

**References**

1. Podsakoff PM, MacKenzie SB, Lee JY, Podsakoff NP. Common method biases in behavioral research: a critical review of the literature and recommended remedies. J Appl Psychol. 2003;88(5):879-903.

2. Van Buuren S, Groothuis-Oudshoorn K. mice: Multivariate imputation by chained equations in R. Journal of statistical software. 2011;45:1-67.

3. Panayiotou M, Black L, Carmichael-Murphy P, Qualter P, Humphrey N. Time spent on social media among the least influential factors in adolescent mental health: preliminary results from a panel network analysis. Nature Mental Health. 2023;1(5):316-26.

4. Epskamp S, Borsboom D, Fried EI. Estimating psychological networks and their accuracy: A tutorial paper. Behav Res Methods. 2018;50(1):195-212.

5. van Borkulo CD, Borsboom D, Epskamp S, Blanken TF, Boschloo L, Schoevers RA, et al. A new method for constructing networks from binary data. Sci Rep. 2014;4:5918.

6. Friedman J, Hastie T, Tibshirani R. Sparse inverse covariance estimation with the graphical lasso. Biostatistics. 2008;9(3):432-41.

7. Foygel R, Drton M. Extended Bayesian information criteria for Gaussian graphical models. Advances in neural information processing systems. 2010;23.

8. Epskamp S, Cramer AO, Waldorp LJ, Schmittmann VD, Borsboom D. qgraph: Network visualizations of relationships in psychometric data. Journal of statistical software. 2012;48:1-18.

9. Bringmann LF, Elmer T, Epskamp S, Krause RW, Schoch D, Wichers M, et al. What do centrality measures measure in psychological networks? J Abnorm Psychol. 2019;128(8):892-903.

10. Robinaugh DJ, Millner AJ, McNally RJ. Identifying highly influential nodes in the complicated grief network. J Abnorm Psychol. 2016;125(6):747-57.

11. Jones PJ, Ma R, McNally RJ. Bridge Centrality: A Network Approach to Understanding Comorbidity. Multivariate Behav Res. 2021;56(2):353-67.

12. Cramer AO, Waldorp LJ, van der Maas HL, Borsboom D. Comorbidity: a network perspective. Behav Brain Sci. 2010;33(2-3):137-50; discussion 50-93.

13. Haslbeck JM, Waldorp LJ. mgm: Estimating time-varying mixed graphical models in high-dimensional data. Journal of Statistical Software. 2020;93:1-46.

14. Haslbeck JMB, Borsboom D, Waldorp LJ. Moderated Network Models. Multivariate Behav Res. 2021;56(2):256-87.

15. Epskamp S, Fried EI. A tutorial on regularized partial correlation networks. Psychol Methods. 2018;23(4):617-34.

16. Lunansky G, Naberman J, van Borkulo CD, Chen C, Wang L, Borsboom D. Intervening on psychopathology networks: Evaluating intervention targets through simulations. Methods. 2022;204:29-37.

17. Marsman M, Borsboom D, Kruis J, Epskamp S, van Bork R, Waldorp LJ, et al. An Introduction to Network Psychometrics: Relating Ising Network Models to Item Response Theory Models. Multivariate Behav Res. 2018;53(1):15-35.

18. Yang X, Fang Y, Wang Y, Liu S, Cai J, Li H, et al. Multidimensional stressors and depressive and anxiety symptoms in adolescents: A network analysis through simulations. J Affect Disord. 2024;347:364-74.

19. Dalege J, Borsboom D, van Harreveld F, van der Maas HLJ. Network Analysis on Attitudes: A Brief Tutorial. Soc Psychol Personal Sci. 2017;8(5):528-37.

20. Wang F, Wang W, Sun Z, Wu Y. Being insulted by parents is the most severe early adverse experience of anxiety in adulthood. J Affect Disord. 2025;369:321-8.

**Table S1** Descriptive statistics and centrality metrics of Children’s Loneliness Scale (CLS) items

|  | Item | Mean ± SD | Probability | Strength |
| --- | --- | --- | --- | --- |
| CLS01 | It’s easy for me to make new friends at school. ^a^ | 2.83 ± 1.35 | 58.74% | 3.94 |
| CLS03 | I have nobody to talk to. | 1.78 ± 1.17 | 23.25% | 3.62 |
| CLS04 | I’m good at working with other children. ^a^ | 3.00 ± 1.32 | 64.13% | 2.96 |
| CLS06 | It’s hard for me to make friends. | 1.62 ± 1.04 | 16.81% | 5.03 |
| CLS08 | I have lots of friends. ^a^ | 2.35 ± 1.33 | 40.76% | 6.36 |
| CLS09 | I feel alone. | 1.64 ± 1.03 | 17.89% | 4.49 |
| CLS10 | I can find a friend when I need one. ^a^ | 3.03 ± 1.34 | 64.25% | 3.44 |
| CLS12 | It’s hard to get other kids to like me. | 1.78 ± 1.13 | 22.52% | 2.66 |
| CLS14 | I don’t have anyone to play with. | 1.61 ± 1.08 | 17.43% | 4.91 |
| CLS16 | I get along with other kids. ^a^ | 2.54 ± 1.34 | 47.63% | 4.92 |
| CLS17 | I feel left out of things. | 1.69 ± 1.06 | 19.40% | 4.38 |
| CLS18 | There’s nobody I can go to when I need help. | 1.63 ± 1.07 | 17.43% | 4.11 |
| CLS21 | I’m lonely. | 1.53 ± 1.03 | 14.62% | 4.89 |
| CLS22 | I am well-liked by the kids in my class. ^a^ | 3.42 ± 1.32 | 75.28% | 3.50 |
| CLS24 | I don’t have any friends. | 1.42 ± 1.00 | 12.65% | 4.18 |

Notes: ^a^ Reverse-coded items have been recoded for consistency. SD = Standard Deviation. Probability represents the likelihood that a participant’s loneliness symptom is categorized as an activated state (response = 1). Strength is presented as raw values without standardization.

**Table S2** Edge weight matrix of the Ising network for children’s loneliness symptoms

| Items | CLS01 | CLS03 | CLS04 | CLS06 | CLS08 | CLS09 | CLS10 | CLS12 | CLS14 | CLS16 | CLS17 | CLS18 | CLS21 | CLS22 | CLS24 |
| --- | --- | --- | --- | --- | --- | --- | --- | --- | --- | --- | --- | --- | --- | --- | --- |
| CLS01 | 0 | 0 | 0.47 | 0.48 | 1.29 | 0 | 0.64 | 0 | 0 | 0.56 | 0 | 0 | 0 | 0.51 | 0 |
| CLS03 | 0 | 0 | 0 | 0.66 | 0.12 | 0.31 | 0 | 0.11 | 1.00 | 0.29 | 0.41 | 0 | 0.08 | 0 | 0.63 |
| CLS04 | 0.47 | 0 | 0 | 0 | 0.44 | 0 | 0.72 | 0 | 0 | 0.80 | 0 | 0 | 0 | 0.53 | 0 |
| CLS06 | 0.48 | 0.66 | 0 | 0 | 0.46 | 0.59 | 0 | 0.53 | 0.41 | 0.23 | 0.45 | 0.14 | 0.19 | 0.37 | 0.53 |
| CLS08 | 1.29 | 0.12 | 0.44 | 0.46 | 0 | 0.31 | 0.85 | 0 | 0.30 | 0.96 | 0 | 0 | 0.43 | 0.83 | 0.37 |
| CLS09 | 0 | 0.31 | 0 | 0.59 | 0.31 | 0 | 0 | 0.52 | 0.29 | 0 | 0.67 | 0.47 | 1.33 | 0 | 0 |
| CLS10 | 0.64 | 0 | 0.72 | 0 | 0.85 | 0 | 0 | 0 | 0 | 0.67 | 0 | 0 | 0 | 0.56 | 0 |
| CLS12 | 0 | 0.11 | 0 | 0.53 | 0 | 0.52 | 0 | 0 | 0.37 | 0 | 0.53 | 0.34 | 0.25 | 0 | 0 |
| CLS14 | 0 | 1.00 | 0 | 0.41 | 0.30 | 0.29 | 0 | 0.37 | 0 | 0.37 | 0.35 | 0.67 | 0.25 | 0 | 0.90 |
| CLS16 | 0.56 | 0.29 | 0.80 | 0.23 | 0.96 | 0 | 0.67 | 0 | 0.37 | 0 | 0 | 0 | 0 | 0.71 | 0.32 |
| CLS17 | 0 | 0.41 | 0 | 0.45 | 0 | 0.67 | 0 | 0.53 | 0.35 | 0 | 0 | 1.06 | 0.60 | 0 | 0.31 |
| CLS18 | 0 | 0 | 0 | 0.14 | 0 | 0.47 | 0 | 0.34 | 0.67 | 0 | 1.06 | 0 | 1.03 | 0 | 0.40 |
| CLS21 | 0 | 0.08 | 0 | 0.19 | 0.43 | 1.33 | 0 | 0.25 | 0.25 | 0 | 0.60 | 1.03 | 0 | 0 | 0.73 |
| CLS22 | 0.51 | 0 | 0.53 | 0.37 | 0.83 | 0 | 0.56 | 0 | 0 | 0.71 | 0 | 0 | 0 | 0 | 0 |
| CLS24 | 0 | 0.63 | 0 | 0.53 | 0.37 | 0 | 0 | 0 | 0.90 | 0.32 | 0.31 | 0.40 | 0.73 | 0 | 0 |

Note: In the Ising network, edge weights represent conditional dependencies between binary variables, estimated as logistic regression coefficients that reflect unique associations between node pairs after controlling for all other variables in the network.

**Table S3** Simulated changes in total loneliness scores in response to alleviating interventions at each node

| Intervention item | Mean Sum score | 95% CI Lower | 95% CI Upper | *t* | *P* |
| --- | --- | --- | --- | --- | --- |
| original | 4.57 | 4.496 | 4.640 | - | - |
| CLS22 | 3.25 | 3.185 | 3.319 | 26.279 | ＜10^-100^ |
| CLS10 | 3.37 | 3.300 | 3.433 | 24.055 | ＜10^-100^ |
| CLS04 | 3.38 | 3.312 | 3.446 | 23.768 | ＜10^-100^ |
| CLS01 | 3.39 | 3.326 | 3.450 | 24.334 | ＜10^-100^ |
| CLS16 | 3.52 | 3.456 | 3.582 | 21.524 | ＜10^-80^ |
| CLS08 | 3.69 | 3.630 | 3.753 | 18.131 | ＜10^-60^ |
| CLS17 | 4.15 | 4.086 | 4.215 | 8.463 | ＜10^-10^ |
| CLS03 | 4.20 | 4.137 | 4.269 | 7.313 | ＜10^-10^ |
| CLS18 | 4.21 | 4.146 | 4.277 | 7.205 | ＜10^-10^ |
| CLS12 | 4.22 | 4.152 | 4.284 | 7.040 | ＜10^-10^ |
| CLS06 | 4.25 | 4.180 | 4.311 | 6.491 | ＜0.001 |
| CLS14 | 4.26 | 4.191 | 4.322 | 6.290 | ＜0.001 |
| CLS21 | 4.27 | 4.203 | 4.333 | 6.070 | ＜0.001 |
| CLS24 | 4.29 | 4.221 | 4.354 | 5.610 | ＜0.001 |
| CLS09 | 4.30 | 4.232 | 4.363 | 5.459 | ＜0.001 |

**Table S4** Simulated changes in total loneliness scores in response to aggravating interventions at each node

| Intervention item | Mean Sum score | 95% CI Lower | 95% CI Upper | *t* | *P* |
| --- | --- | --- | --- | --- | --- |
| original | 4.56 | 4.490 | 4.635 | - | - |
| CLS08 | 6.12 | 6.052 | 6.194 | -30.125 | ＜10^-150^ |
| CLS06 | 5.88 | 5.790 | 5.967 | -22.559 | ＜10^-100^ |
| CLS14 | 5.86 | 5.773 | 5.946 | -22.544 | ＜10^-100^ |
| CLS16 | 5.82 | 5.753 | 5.891 | -24.713 | ＜10^-120^ |
| CLS21 | 5.81 | 5.727 | 5.901 | -21.667 | ＜10^-80^ |
| CLS09 | 5.79 | 5.703 | 5.872 | -21.641 | ＜10^-80^ |
| CLS18 | 5.77 | 5.689 | 5.858 | -21.297 | ＜10^-80^ |
| CLS03 | 5.76 | 5.676 | 5.841 | -21.379 | ＜10^-80^ |
| CLS17 | 5.71 | 5.629 | 5.795 | -20.423 | ＜10^-80^ |
| CLS24 | 5.66 | 5.575 | 5.748 | -19.134 | ＜10^-60^ |
| CLS12 | 5.63 | 5.550 | 5.713 | -19.209 | ＜10^-60^ |
| CLS01 | 5.51 | 5.446 | 5.580 | -18.904 | ＜10^-60^ |
| CLS10 | 5.43 | 5.357 | 5.493 | -17.070 | ＜10^-60^ |
| CLS04 | 5.34 | 5.269 | 5.404 | -15.303 | ＜10^-40^ |
| CLS22 | 5.17 | 5.107 | 5.242 | -12.121 | ＜10^-20^ |

Notes for Table S3 and S4: Multiple independent samples *t*-tests were conducted to compare each simulated intervention condition with the original level of loneliness. *P*-values were adjusted using the Bonferroni correction for multiple comparisons. CI = Confidence Interval.

**Table S5** Descriptive statistics and centrality metrics of loneliness and related psychological variables in the domain-level Graphical Gaussian Model (GGM)

| Variable | Description | Mean ± SD | Strength | Expected Influence |
| --- | --- | --- | --- | --- |
| CLS | Children’s loneliness | 33.41 ± 9.98 | 1.04 | 0.14 |
| DSRS | Children’s depressive symptoms | 12.70 ± 5.44 | 0.81 | 0.03 |
| SCARED | Children’s anxiety symptoms | 13.87 ± 11.27 | 0.57 | 0.47 |
| ASQ | ADHD symptoms | 4.82 ± 4.47 | 0.22 | 0.20 |
| PSSS | Perceived social support | 58.87 ± 16.82 | 0.76 | -0.09 |
| CHS | Children’s hope | 21.22 ± 6.65 | 0.87 | -0.09 |

Notes: SD = Standard Deviation. Centrality metrics (strength and expected influence) are reported in their raw (non-standardized) form.

**Table S6** Edge weight matrix of loneliness and related psychological variables in the domain-level GGM

| Variable | CLS | DSRS | SCARED | ASQ | PSSS | CHS |
| --- | --- | --- | --- | --- | --- | --- |
| CLS | 0 | 0.29 | 0.25 | 0.05 | -0.22 | -0.23 |
| DSRS | 0.29 | 0 | 0.09 | 0.04 | -0.15 | -0.24 |
| SCARED | 0.25 | 0.09 | 0 | 0.13 | -0.05 | 0.06 |
| ASQ | 0.05 | 0.04 | 0.13 | 0 | 0 | -0.01 |
| PSSS | -0.22 | -0.15 | -0.05 | 0 | 0 | 0.33 |
| CHS | -0.23 | -0.24 | 0.06 | -0.01 | 0.33 | 0 |

Note: In the GGM, edge weights represent partial correlation coefficients between continuous variables, controlling for all other variables in the network.

**Table S7** Descriptive statistics and bridge centrality metrics of loneliness and related psychological variables in the facet-level GGM

| Variable | Description | Mean ± SD | Bridge Strength | Bridge Expected Influence (1-step) | Bridge Expected Influence (2-step) |
| --- | --- | --- | --- | --- | --- |
| CLS | Children’s loneliness | 33.41 ± 9.98 | 0.79 | -0.11 | -0.29 |
| DSRS_1 | Children’s depressive symptoms-Low positive affect | 9.63 ± 4.72 | 0.70 | -0.70 | -1.18 |
| DSRS_2 | Children’s depressive symptoms-Negative affect | 3.07 ± 2.82 | 0.35 | 0.12 | 0.08 |
| SCARED_1 | Children’s anxiety symptoms-Somatic/panic | 3.46 ± 3.67 | 0.04 | -0.01 | 0.02 |
| SCARED_2 | Children’s anxiety symptoms-General anxiety | 2.94 ± 3.10 | 0.15 | 0.02 | 0.00 |
| SCARED_3 | Children’s anxiety symptoms-Separation anxiety | 3.05 ± 2.86 | 0.10 | 0.01 | 0.01 |
| SCARED_4 | Children’s anxiety symptoms-Social phobia | 3.24 ± 2.75 | 0.19 | -0.11 | -0.11 |
| SCARED_5 | Children’s anxiety symptoms-School phobia | 1.18 ± 1.30 | 0.11 | -0.01 | -0.03 |
| ASQ | ADHD symptoms | 4.82 ± 4.47 | 0.14 | 0.12 | 0.21 |
| PSSS_1 | Perceived social support-Family | 20.06 ± 5.91 | 0.30 | -0.01 | -0.06 |
| PSSS_2 | Perceived social support-Friends | 19.46 ± 6.09 | 0.24 | -0.18 | -0.20 |
| PSSS_3 | Perceived social support-Significant Other | 19.35 ± 6.35 | 0.21 | 0.01 | -0.08 |
| CHS_1 | Children’s hope-Agency | 10.32 ± 3.52 | 0.53 | -0.34 | -0.32 |
| CHS_2 | Children’s hope-Pathways | 10.90 ± 3.70 | 0.36 | 0.04 | -0.02 |

Notes: SD = Standard Deviation. Bridge centrality metrics (bridge strength and bridge expected influence [1-step and 2-step]) are reported in their raw (non-standardized) form.

**Table S8** Edge weight matrix of loneliness and related psychological variables in the facet-level GGM

| Variable | CLS | DSRS_1 | DSRS_2 | SCARED_1 | SCARED_2 | SCARED_3 | SCARED_4 | SCARED_5 | ASQ | PSSS_1 | PSSS_2 | PSSS_3 | CHS_1 | CHS_2 |
| --- | --- | --- | --- | --- | --- | --- | --- | --- | --- | --- | --- | --- | --- | --- |
| CLS | 0 | 0.19 | 0.20 | 0 | 0.08 | 0.01 | 0.04 | 0 | 0.05 | 0 | -0.16 | -0.05 | -0.17 | -0.08 |
| DSRS_1 | 0.19 | 0 | -0.08 | -0.03 | -0.04 | -0.04 | -0.09 | 0 | 0 | -0.07 | -0.05 | -0.01 | -0.21 | -0.08 |
| DSRS_2 | 0.20 | -0.08 | 0 | 0.20 | 0.19 | 0.07 | 0.02 | 0.22 | 0.02 | -0.03 | 0 | 0 | 0 | 0.01 |
| SCARED_1 | 0 | -0.03 | 0.20 | 0 | 0.35 | 0.20 | 0.13 | 0.18 | 0.02 | 0 | 0 | 0 | 0 | 0 |
| SCARED_2 | 0.08 | -0.04 | 0.19 | 0.35 | 0 | 0.28 | 0.20 | 0.02 | 0.01 | -0.02 | 0 | 0 | -0.01 | 0 |
| SCARED_3 | 0.01 | -0.04 | 0.07 | 0.20 | 0.28 | 0 | 0.20 | 0.10 | 0.04 | 0 | 0 | 0 | 0 | 0 |
| SCARED_4 | 0.04 | -0.09 | 0.02 | 0.13 | 0.20 | 0.20 | 0 | 0 | 0 | 0 | 0 | -0.02 | -0.04 | 0 |
| SCARED_5 | 0 | 0 | 0.22 | 0.18 | 0.02 | 0.10 | 0 | 0 | 0.05 | -0.02 | 0 | -0.03 | 0 | 0 |
| ASQ | 0.05 | 0 | 0.02 | 0.02 | 0.01 | 0.04 | 0 | 0.05 | 0 | 0 | 0 | 0 | -0.01 | 0 |
| PSSS_1 | 0 | -0.07 | -0.03 | 0 | -0.02 | 0 | 0 | -0.02 | 0 | 0 | 0.26 | 0.41 | 0.06 | 0.08 |
| PSSS_2 | -0.16 | -0.05 | 0 | 0 | 0 | 0 | 0 | 0 | 0 | 0.26 | 0 | 0.44 | 0.01 | 0.02 |
| PSSS_3 | -0.05 | -0.01 | 0 | 0 | 0 | 0 | -0.02 | -0.03 | 0 | 0.41 | 0.44 | 0 | 0.02 | 0.09 |
| CHS_1 | -0.17 | -0.21 | 0 | 0 | -0.01 | 0 | -0.04 | 0 | -0.01 | 0.06 | 0.01 | 0.02 | 0 | 0.48 |
| CHS_2 | -0.08 | -0.08 | 0.01 | 0 | 0 | 0 | 0 | 0 | 0 | 0.08 | 0.02 | 0.09 | 0.48 | 0 |

Note: In the GGM, edge weights represent partial correlation coefficients between continuous variables, controlling for all other variables in the network.


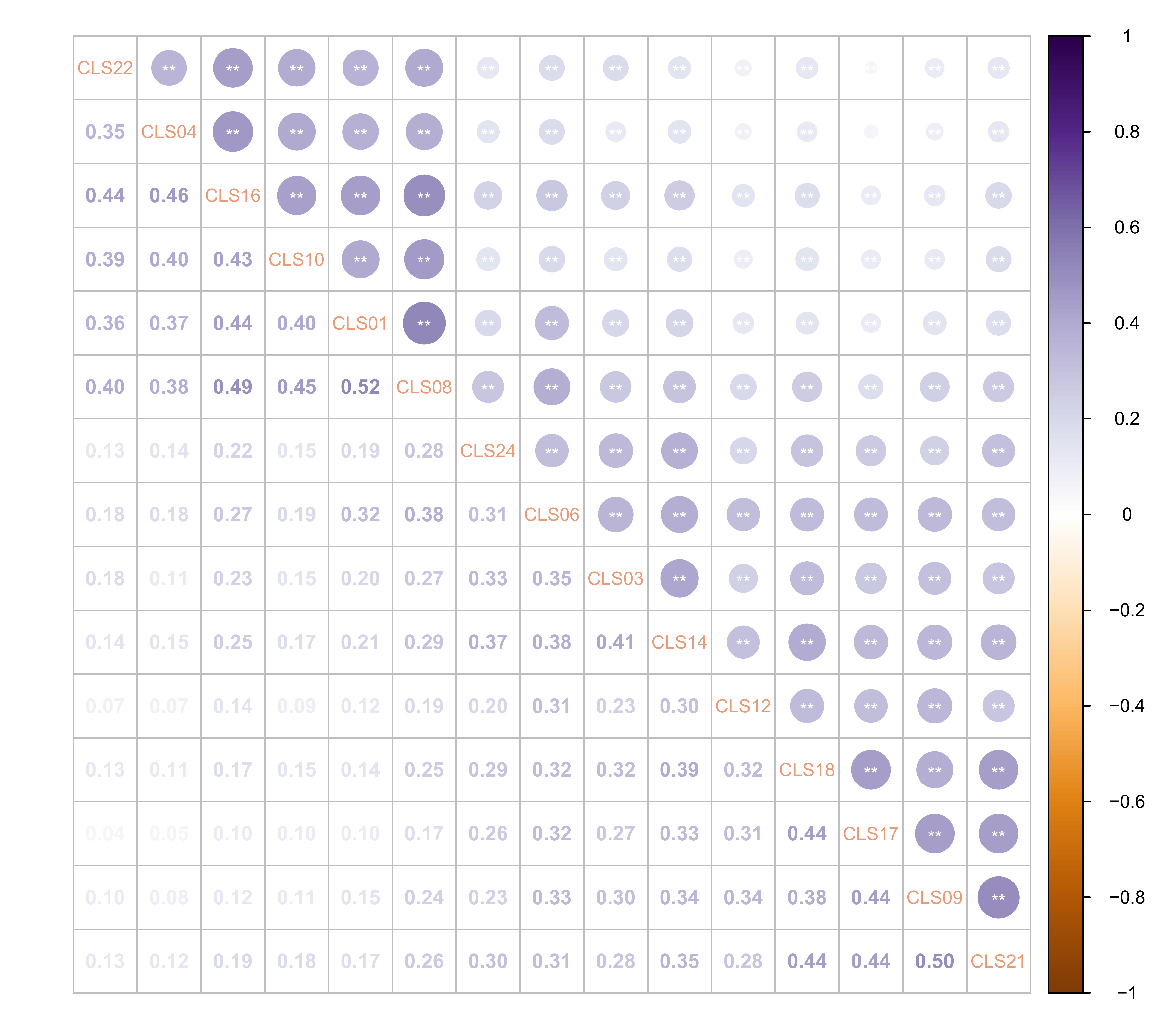


**Fig. S1.** Spearman’s correlation matrix of children’s loneliness symptoms


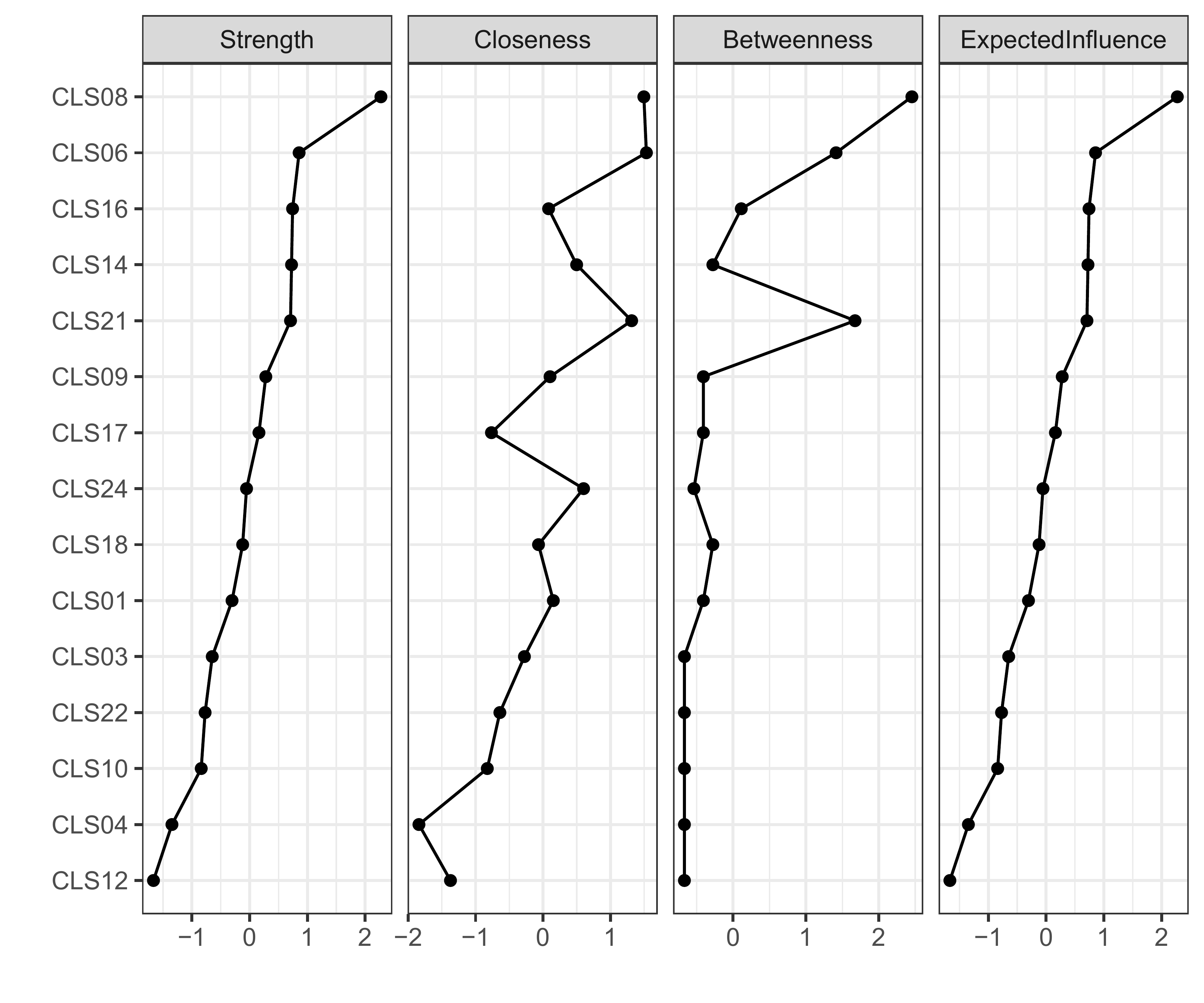


**Fig. S2.** Centrality metrics of children’s loneliness symptoms in the Ising network (presented as z-scores)

Note: Expected influence values are shown for completeness, though they are numerically identical to strength due to the absence of negative edges.


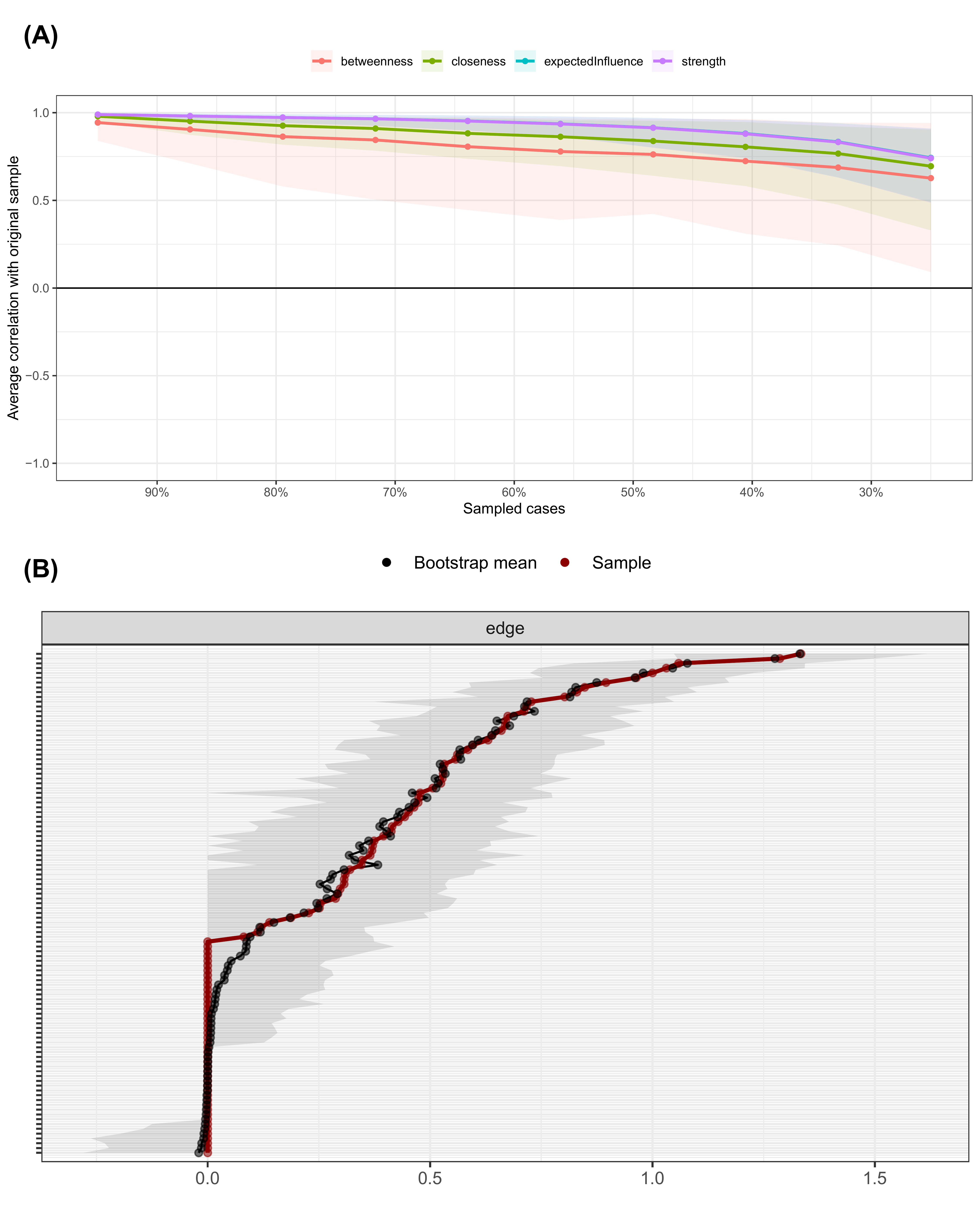


**Fig. S3.** Stability and accuracy analysis of the Ising network

(A) Stability of centrality metrics (strength, closeness, betweenness, expected influence). Strength and expected influence showed strong stability (CS coefficient = 0.59 > 0.50), closeness was acceptable (CS coefficient = 0.44 > 0.25), while betweenness was unstable (CS coefficient = 0.13 < 0.25).

(B) Accuracy of edge weights. Red dots show original edge weights; black dots and gray areas represent bootstrap estimates and 95% CIs. Narrower intervals indicate higher accuracy.


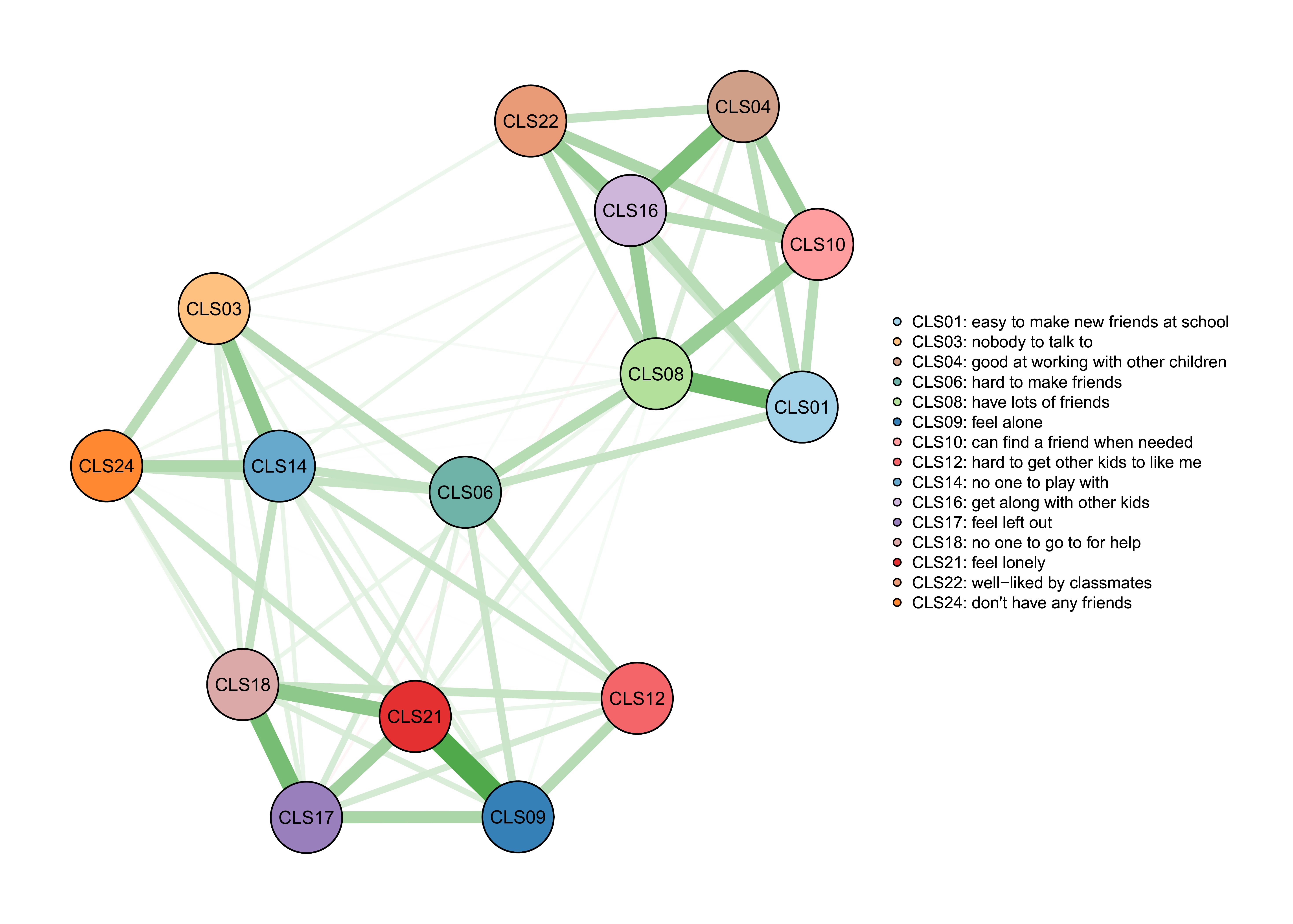


**Fig. S4.** Graphical Gaussian Model (GGM) of loneliness symptoms in school-age children


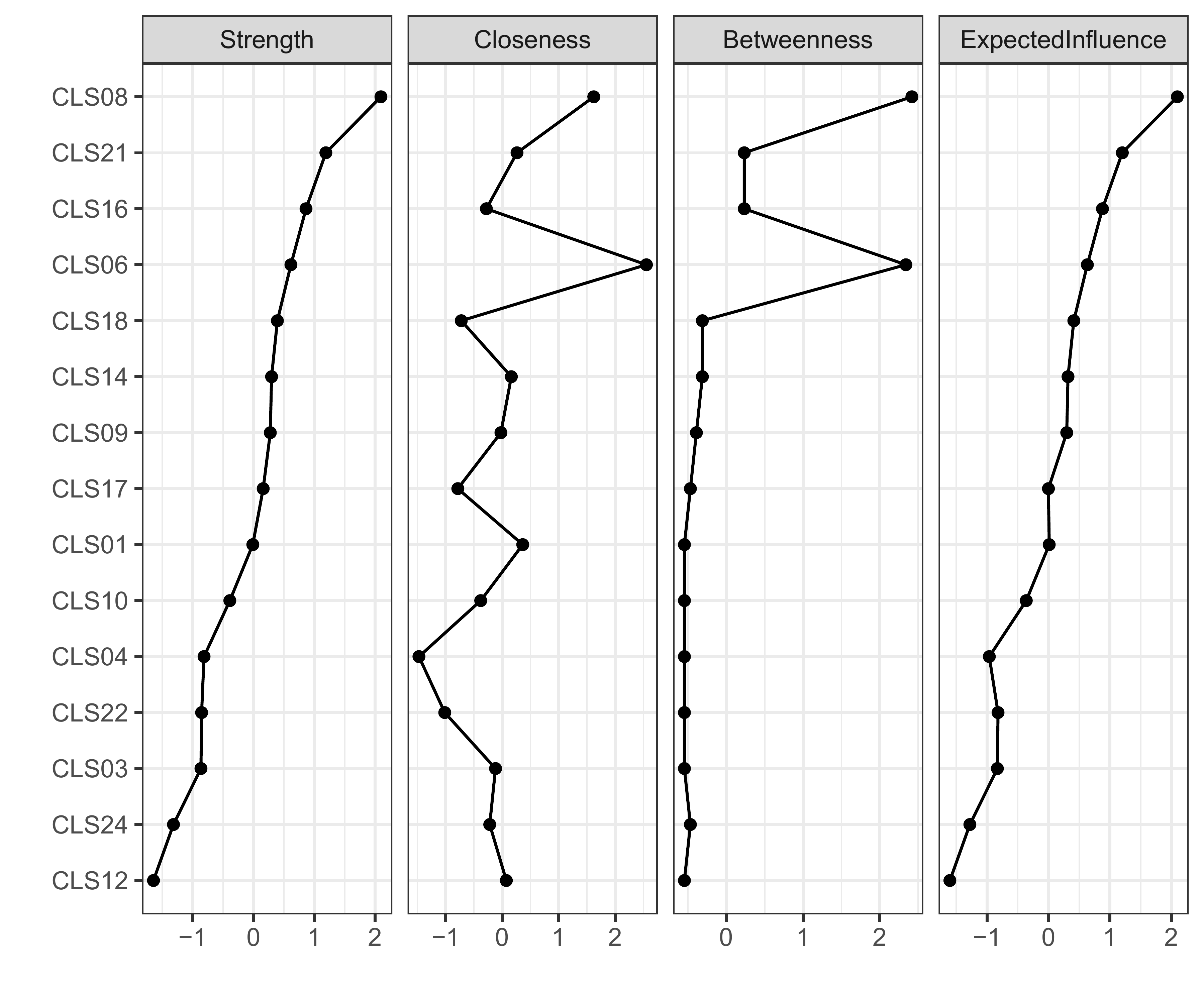


**Fig. S5.** Centrality metrics of children’s loneliness symptoms in the GGM (presented as z-scores)


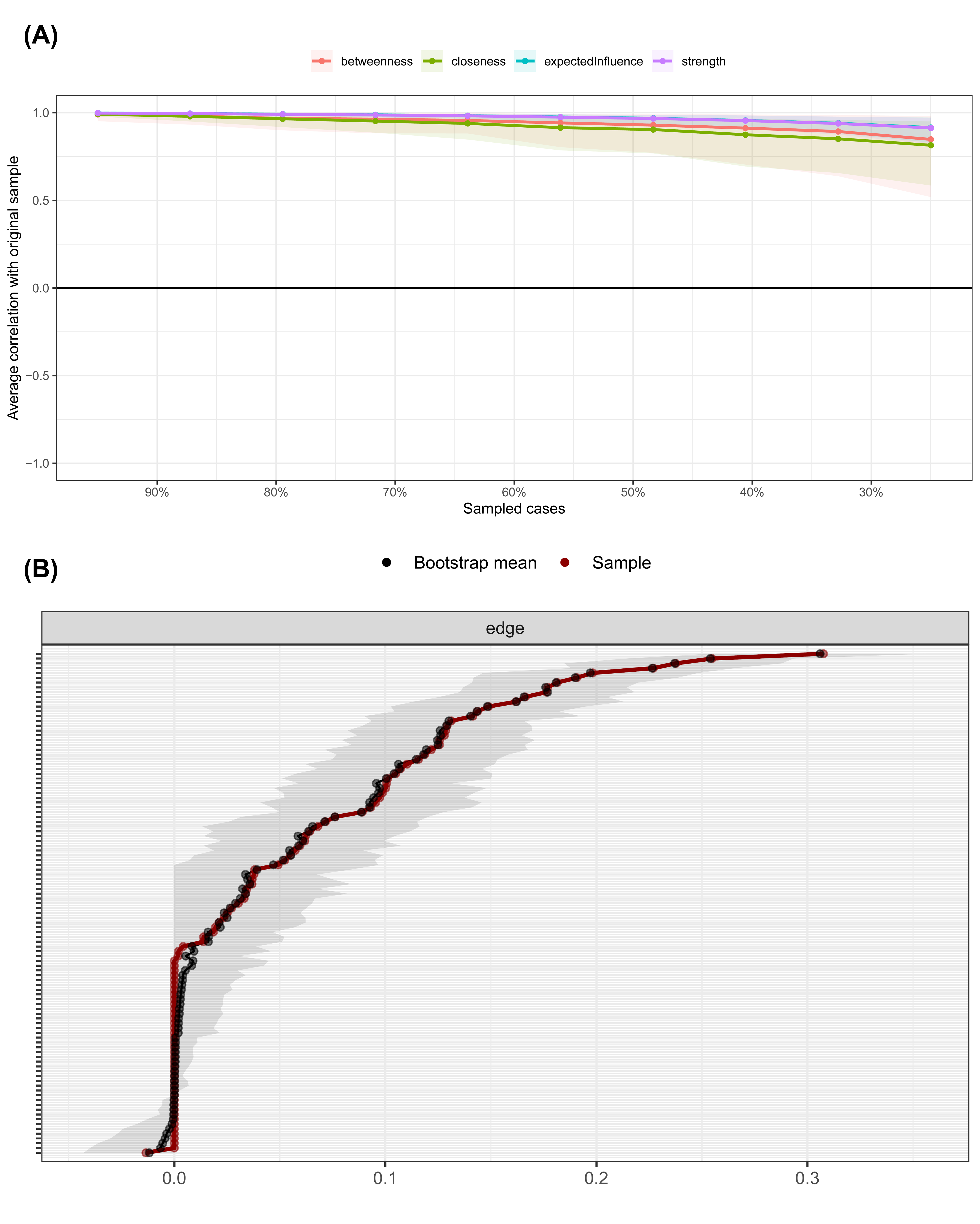


**Fig. S6.** Stability and accuracy analysis of the GGM

(A) Stability of centrality metrics (strength, closeness, betweenness, expected influence). Strength (CS coefficient = 0.75), expected influence (CS coefficient = 0.75), betweenness (CS coefficient = 0.67) and closeness (CS coefficient = 0.59) all showed strong stability (CS coefficient > 0.50).

(B) Accuracy of edge weights. Red dots show original edge weights; black dots and gray areas represent bootstrap estimates and 95% CIs. Narrower intervals indicate higher accuracy.


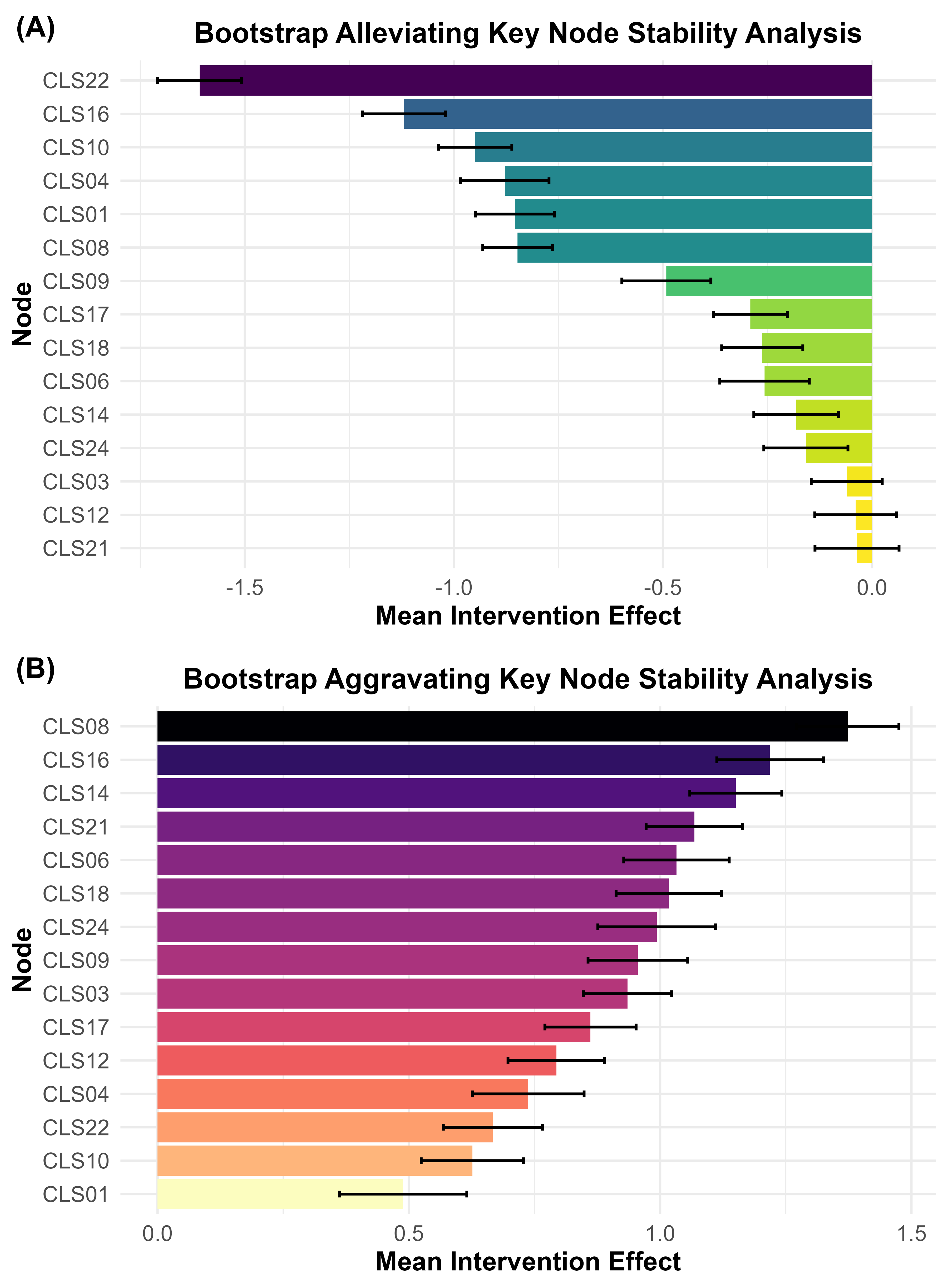


**Fig. S7.** Bootstrap analysis of key node stability under alleviating (A) and aggravating (B) interventions
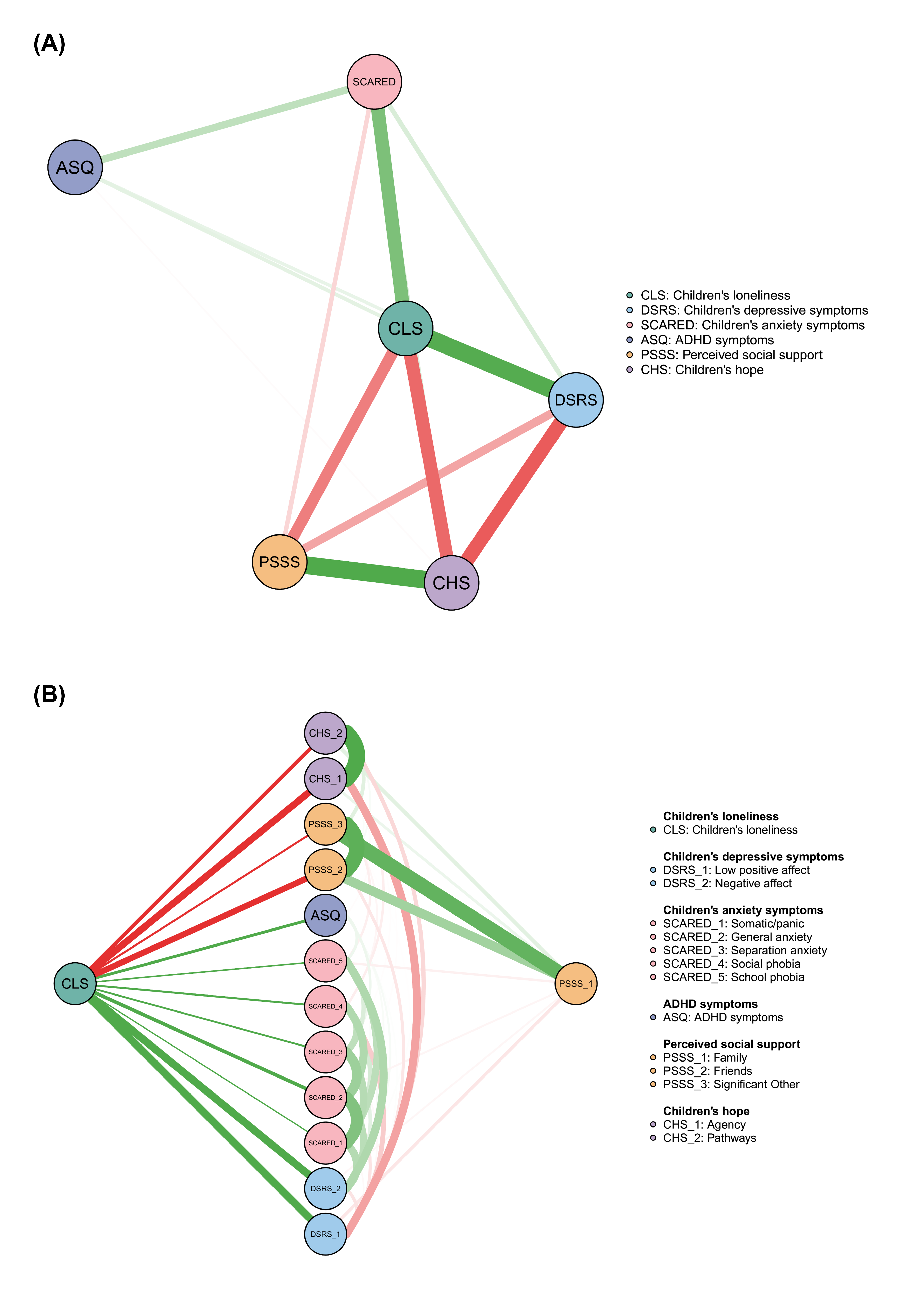
 **Fig. S8.** The relationships between children’s loneliness and related psychological variables in the domain-level (A) and facet-level (B) GGM (no covariates controlled)


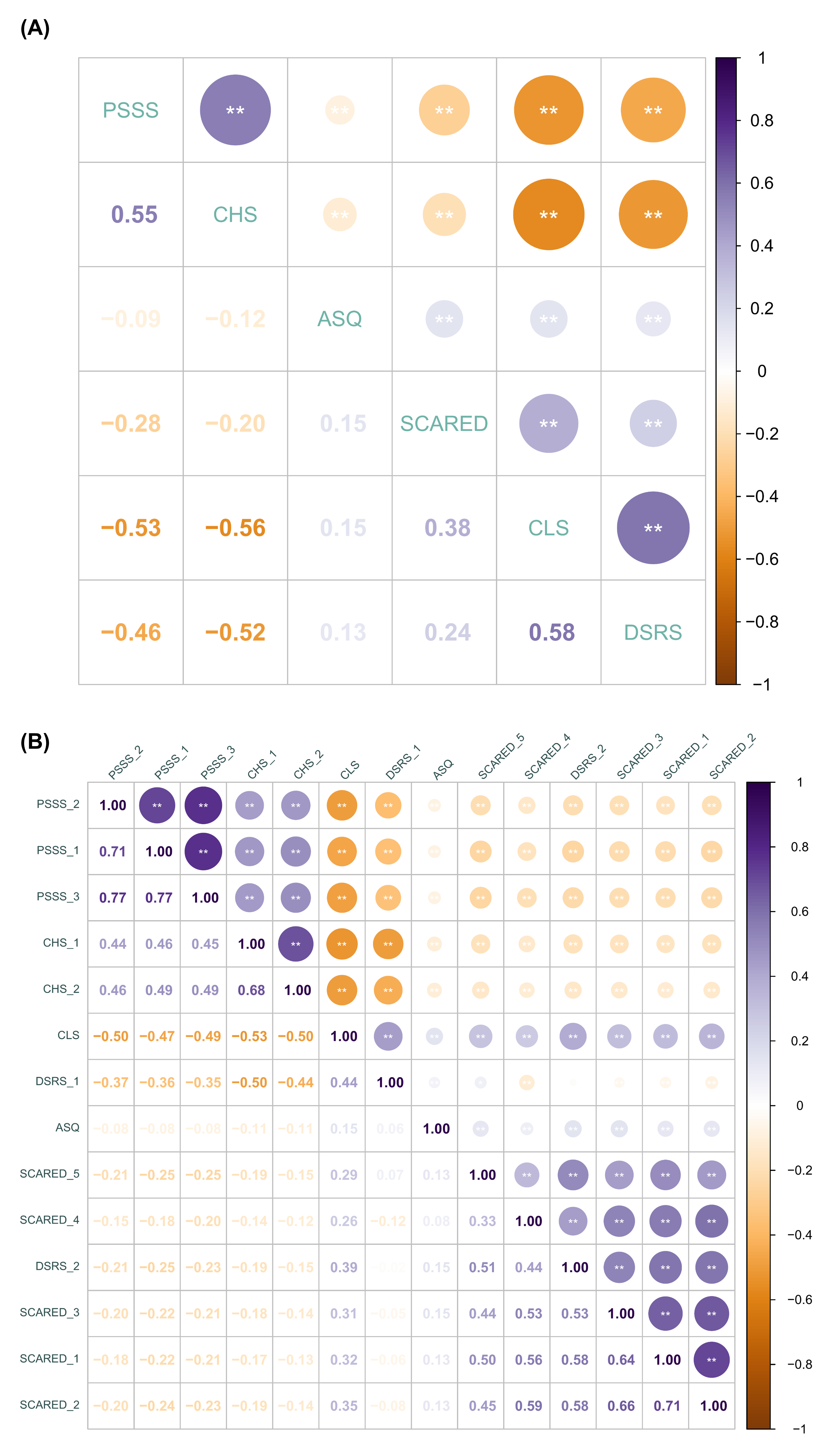


**Fig. S9.** Spearman’s correlation matrix of children’s loneliness and related psychological variables in the domain-level (A) and facet-level (B) GGM


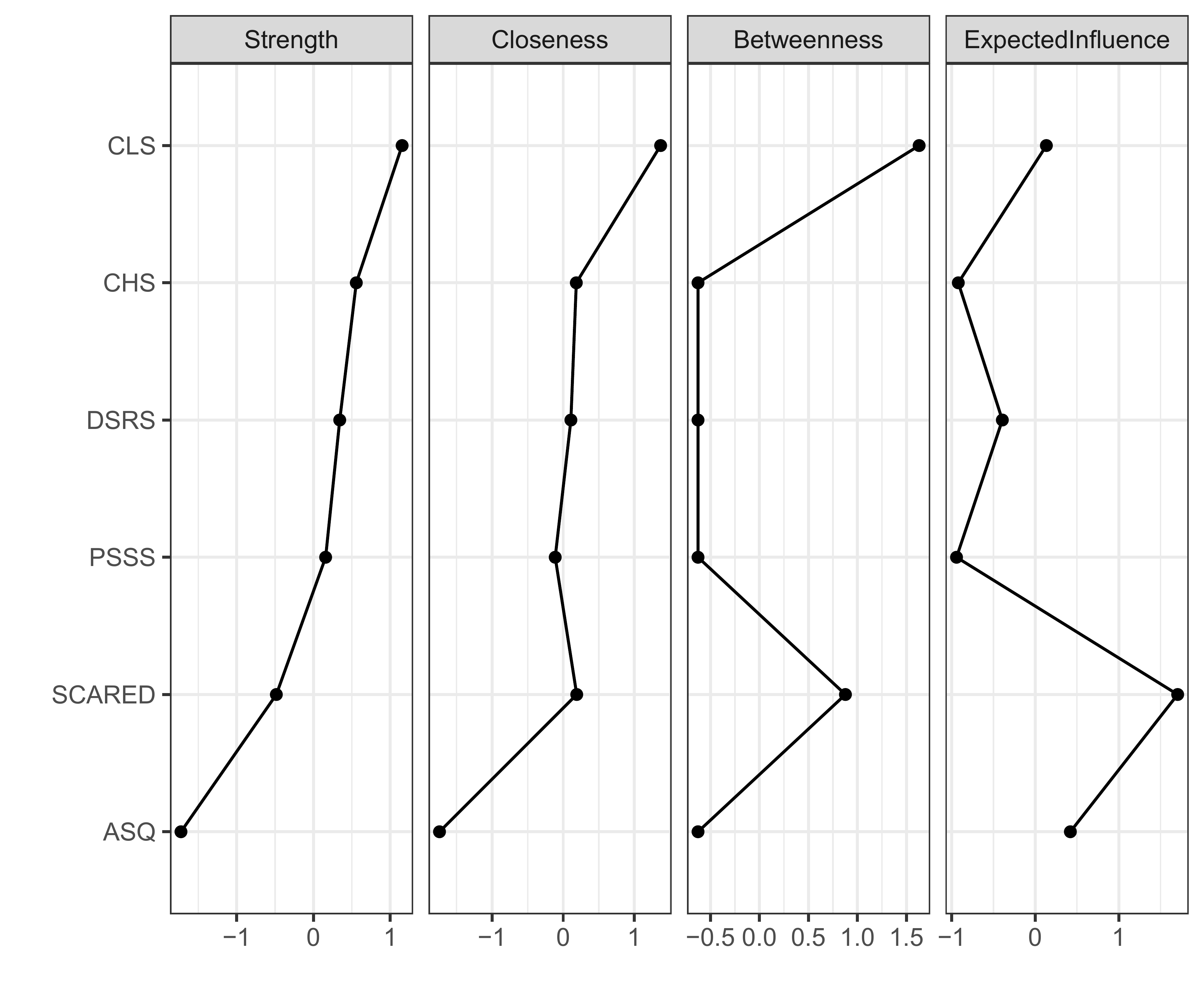


**Fig. S10.** Centrality metrics of children’s loneliness and related psychological variables in the domain-level GGM (presented as z-scores)


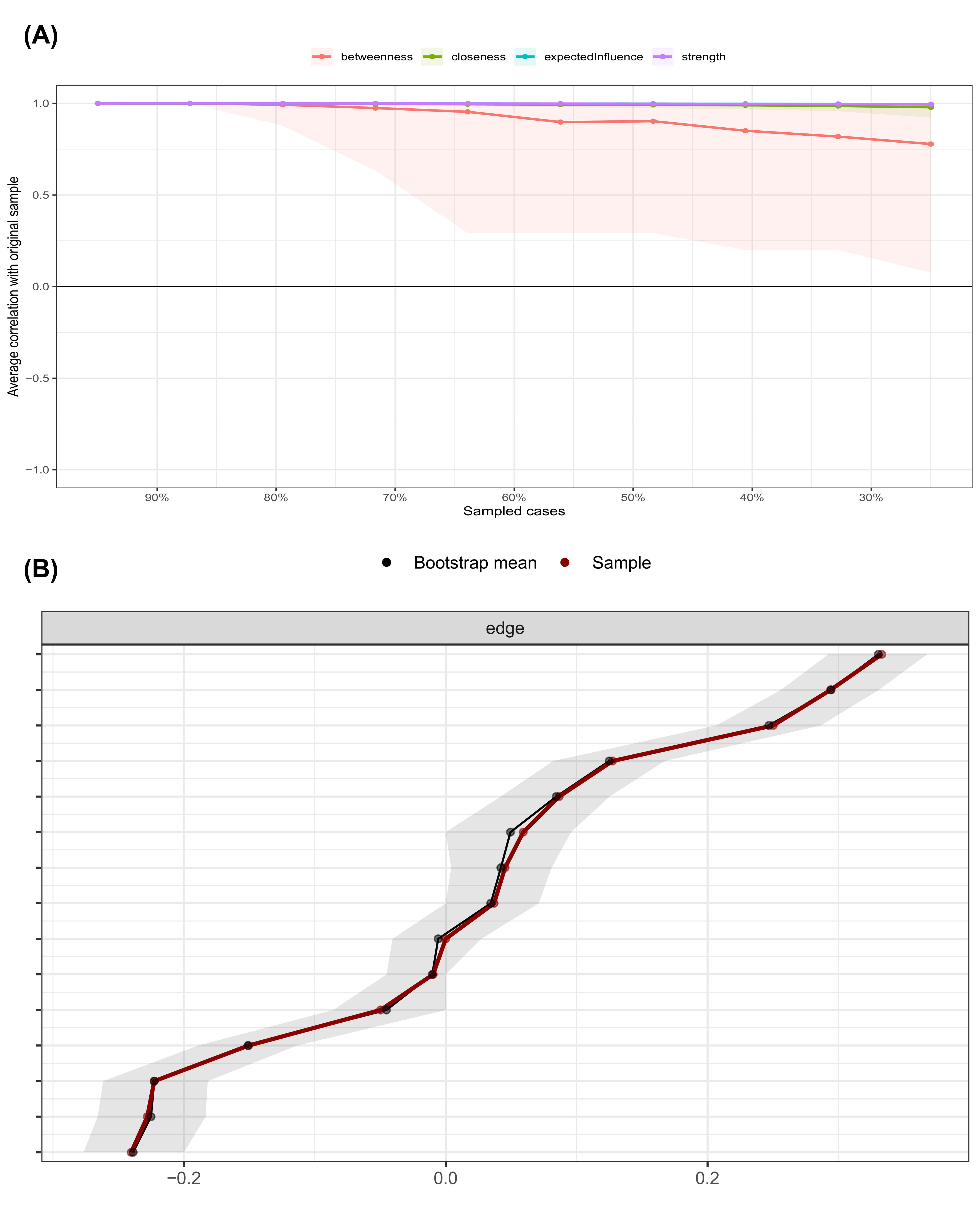


**Fig. S11.** Stability and accuracy of the domain-level GGM for loneliness and related psychological variables

(A) Stability of centrality metrics (strength, closeness, betweenness, expected influence). Strength, expected influence, and closeness showed strong stability (CS coefficient = 0.75 > 0.50), while betweenness showed moderate stability (CS coefficient = 0.28 > 0.25).

(B) Accuracy of edge weights. Red dots show original edge weights; black dots and gray areas represent bootstrap estimates and 95% CIs. Narrower intervals indicate higher accuracy.


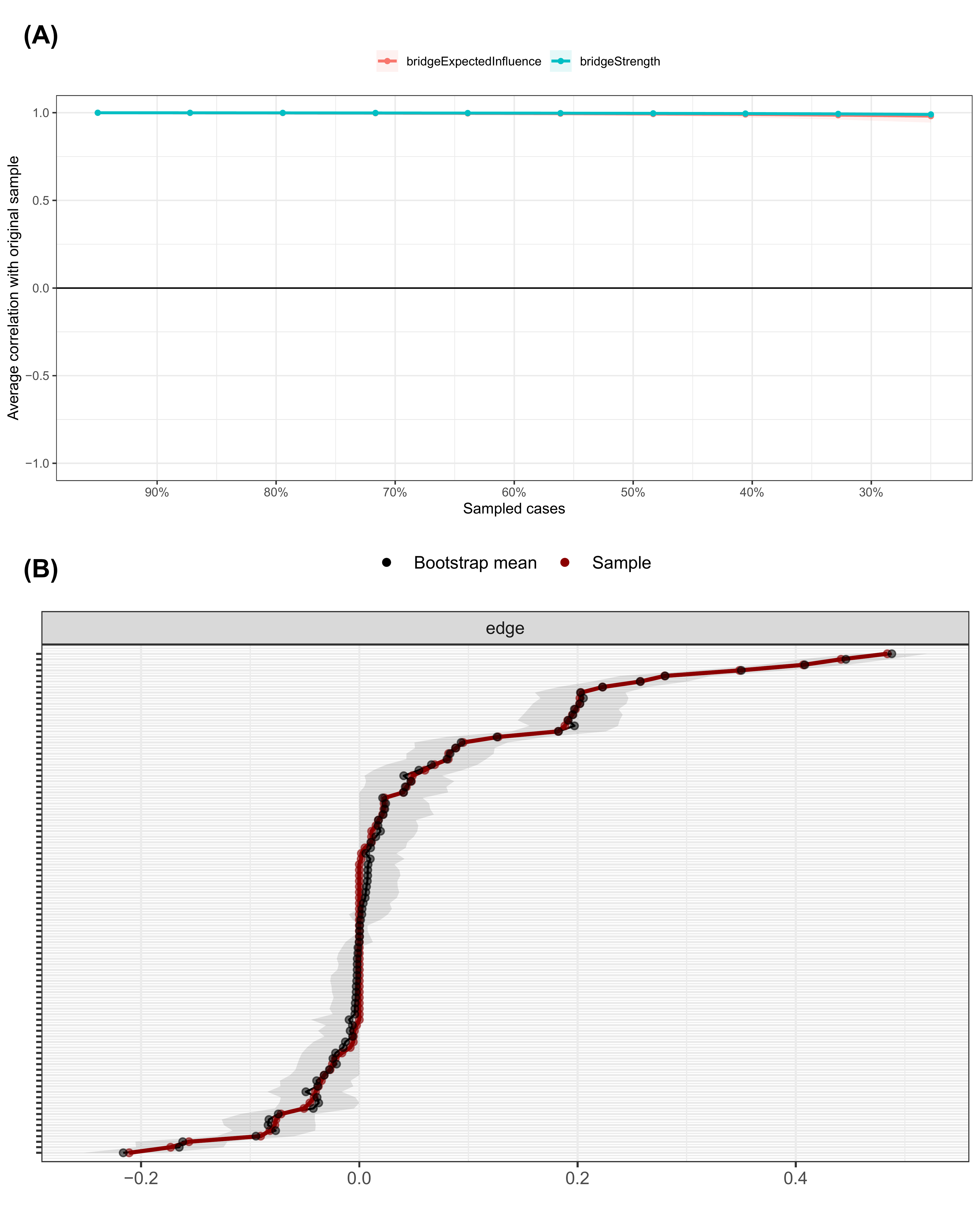


**Fig. S12.** Stability and accuracy of the facet-level GGM for loneliness and related psychological variables

(A) Stability of bridge centrality metrics (bridge strength, bridge expected influence). Bridge strength and bridge expected influence both showed strong stability (CS coefficient = 0.75 > 0.50).

(B) Accuracy of edge weights. Red dots show original edge weights; black dots and gray areas represent bootstrap estimates and 95% CIs. Narrower intervals indicate higher accuracy.


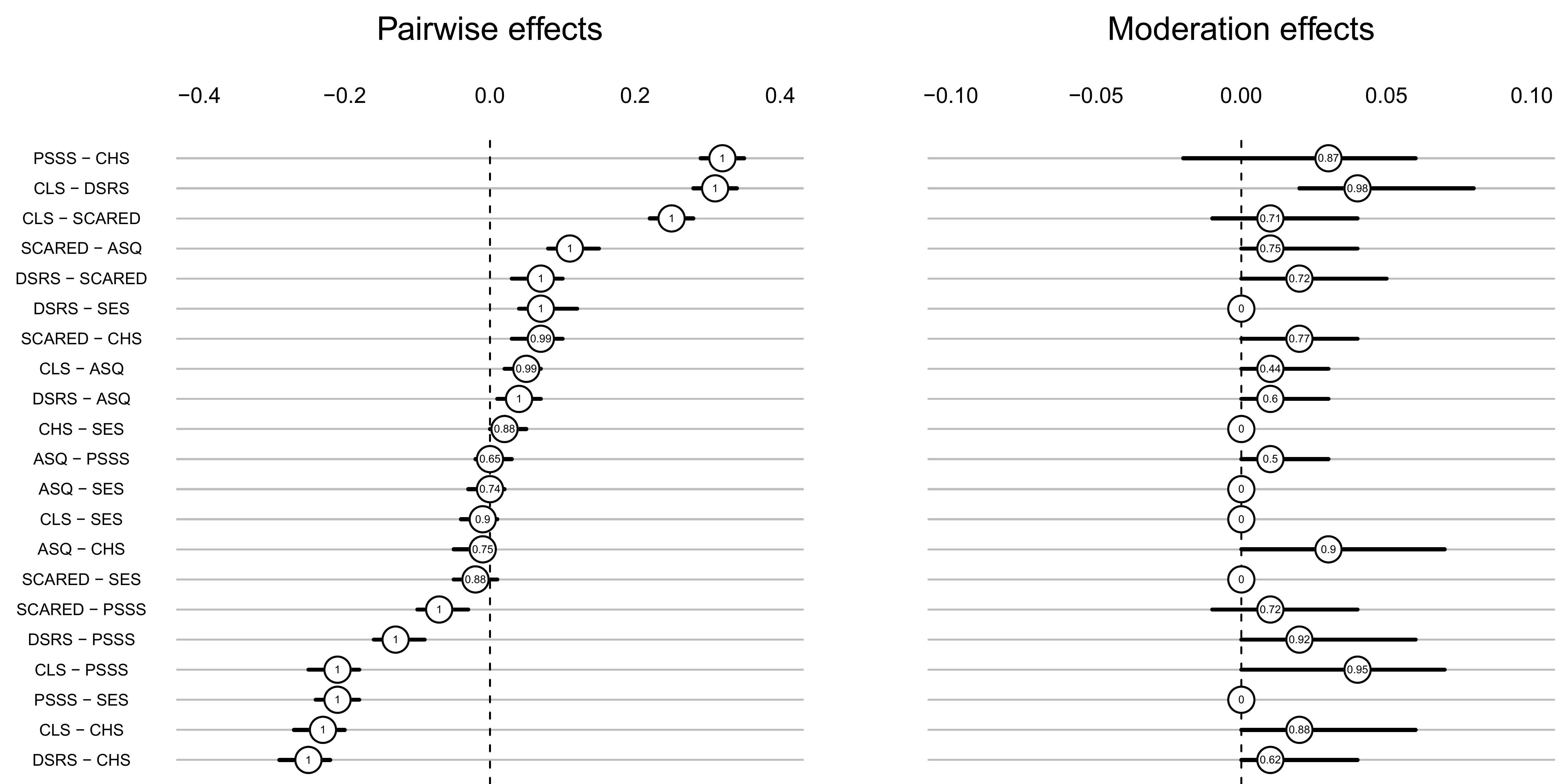


**Fig. S13.** Bootstrapped sampling distributions in the Moderated Network Model

Notes: The left panel displays pairwise effects; the right, moderation effects. Values indicate the proportion of bootstrap samples with nonzero estimates. Horizontal lines show the 5^th^ and 95^th^ percentiles; value positions represent mean estimates. CLS = Children’s loneliness; DSRS = Children’s depressive symptoms; SCARED = Children’s anxiety symptoms; ASQ = ADHD symptoms; PSSS = Perceived social support; CHS = Children’s hope; SES = Family socioeconomic status.
